# Integrated Transcriptomic and Proteomic Analysis Unveils the Multi-Organ Regulatory Mechanisms of Growth Divergence in Grass Carp (*Ctenopharyngodon idella*)

**DOI:** 10.3390/ani16142205

**Published:** 2026-07-15

**Authors:** Tengfei Zhu, Hao Chen, Zhipeng Zheng, Huayang Guo, Baosuo Liu, Kecheng Zhu, Nan Zhang, Lin Xian, Yingying Yu, Yang Liu, Songlin Chen, Dianchang Zhang

**Affiliations:** 1 Key Laboratory of South China Sea Fishery Resources Exploitation and Utilization, Ministry of Agriculture and Rural Affairs, South China Sea Fisheries Research Institute, Chinese Academy of Fishery Sciences, Guangzhou 510300, China; 2 Sanya Tropical Fisheries Research Institute, Sanya 572018, China; 3 Guangdong Provincial Key Laboratory of Animal Molecular Design and Precise Breeding, School of Animal Science and Technology, Foshan University, Foshan 528225, China; 4 Guangdong Engineering Research Center of Key Technologies and Equipment R&D on Modern Marine Ranching, Guangzhou 510300, China; 5 Guangdong Provincial Engineer Technology Research Center of Marine Biological Seed Industry, Guangzhou 510300, China; 6 State Key Laboratory of Mariculture Biobreeding and Sustainable Goods, Yellow Sea Fisheries Research Institute, Chinese Academy of Fishery Sciences, Qingdao 266000, China

**Keywords:** grass carp, transcriptomics, proteomics, growth performance, energy metabolism

## Abstract

Grass carp (*Ctenopharyngodon idella*) is an important freshwater aquaculture species in China. However, high-density farming often leads to significant growth differentiation among individuals, seriously affecting yield and product quality. Here, we conducted an integrated transcriptomic and data-independent acquisition (DIA) proteomic analysis across the brain, liver, and muscle tissues of fast-growing (FG) and slow-growing (SG) grass carp after 9 months of high-density culture. Our analysis revealed that the core mechanism driving growth differentiation is a deep decoupling of transcription and translation, where enhanced muscle translational efficiency dictates the fast-growth phenotype, while slow growth is constrained by central stress and hepatic energy depletion. In the slow growth group, the brain exhibited translational arrest and neuroinflammation, and the liver entered a state of hypermetabolism mediated by AMPK, evidenced by the post-transcriptional up-regulation of key sensors such as CAB39 (PRM ratio = 1.634) and CAMKK2 (PRM ratio = 1.295). Conversely, in the fast-growing group, the mTOR–ribosome signaling axis was strongly activated at the post-transcriptional level in the muscle (GSEA NES = −1.852), triggering myofibrillar protein deposition despite transcriptional silence in classical growth pathways. These findings elucidate the systemic molecular mechanisms of growth divergence and provide high-confidence molecular targets for developing fast-growing, stress-resilient grass carp strains for precision aquaculture breeding.

## Simple Summary

Grass carp is an important freshwater aquaculture species in China. However, high-density farming often leads to significant growth differentiation in ponds, causing severe economic losses. To explore why some individuals grow rapidly while others grow slowly in high-density artificial farming environments, we conducted a study on the brain, liver, and muscle tissues of fast-growing and slow-growing grass carp in high-density farming conditions. The research found that slow-growing individuals have a stress response in the brain and an overloaded liver metabolism, resulting in the consumption of essential nutrients and the failure to convert them into body growth. In contrast, fast-growing individuals maintain an efficient metabolic state in the liver and directly promote protein synthesis in the muscle tissue, thereby achieving rapid muscle accumulation. This study indicates that rapid growth depends on efficient nutrient delivery and muscle protein deposition, while slow growth is the consequence of systemic stress and energy depletion. These findings are helpful in identifying specific biological markers for the selection and breeding of stress-resistant and fast-growing superior varieties that are adapted to modern aquaculture models, ultimately improving the growth efficiency of fish in high-density farming.

## 1. Introduction

Grass carp (*Ctenopharyngodon idella*) is currently a common freshwater aquaculture species in the world, playing an indispensable role in ensuring a high-quality protein supply [1,2]. With the rapid modernization and intensification of the aquaculture industry, high-density farming has become the standard practice for maximizing space utilization and economic output. However, the high-density farming environment triggers a series of environmental stressors, including long-term crowding, deteriorating water quality, ammonia nitrogen accumulation, and intense social hierarchy competition for food [3,4]. Under such intensive farming conditions, significant growth differentiation is often observed among individuals and even within full-sibling families. This growth disparity leads to inconsistent product sizes of adult fish, a significant reduction in feed conversion rate, and affects product quality while causing economic losses to the aquaculture industry [5]. This phenomenon indicates that the growth performance of bony fish is not only a result of rearing conditions but is determined by a comprehensive balance of energy within the animal and physiological and stress adaptation mechanisms [6,7].

In teleost fish, growth is not an isolated trait restricted to a single organ but a systemic process orchestrated by the coordinated regulation of multi-organ axes, such as the “brain-liver-muscle” axis [8,9]. Within this regulatory network, the hypothalamic–pituitary system integrates external environmental cues and internal nutritional signals to modulate the canonical growth hormone/insulin-like growth factor-1 (GH/IGF-1) axis [10,11]. This neuroendocrine cascade exerts precise control over systemic nutrient allocation and skeletal muscle development. Skeletal muscle accounts for the largest proportion of fish body weight and serves as the primary site for protein deposition [12]. Unlike mammals, fish muscle growth exhibits high plasticity, with its development relying not only on the proliferation of new muscle fibers (hyperplasia) but also on the enlargement of existing muscle fibers (hypertrophy) [13]. Fundamentally, the sustained expansion of muscle tissue depends on a precise dynamic equilibrium among protein synthesis, ribosome biogenesis, and protein turnover [14].

The sustained expansion of skeletal muscle tissue is fundamentally dependent on the response of myocytes to upstream growth-promoting signals. Within myocytes, the mechanistic target of rapamycin (mTOR) pathway acts as a central hub that links upstream signals to downstream anabolic processes. It can sense systemic neuroendocrine cues (e.g., GH/IGF-1) and the nutritional status of the local microenvironment (e.g., amino acid concentrations). Upon activation, mTOR directly phosphorylates key targets including p70S6 kinase (S6K1) and eukaryotic translation initiation factor 4E-binding protein 1 (4E-BP1), thereby triggering ribosome biogenesis, relieving translational repression, and accelerating protein deposition [15,16]. Thus, maintaining the active state of the mTOR pathway to support translation and protein deposition forms the molecular basis for muscle growth in teleost fish.

Meanwhile, the liver orchestrates the metabolism of carbohydrates, lipids, and amino acids, serving as a central regulator for the supply and allocation of nutrient substrates to support peripheral muscle growth [9,17]. However, chronic stress and nutritional constraints associated with intensive aquaculture can profoundly disrupt hepatic energy homeostasis. When intracellular ATP is depleted or excessive oxidative stress ensues, the cellular energy sensor AMP-activated protein kinase (AMPK) is rapidly activated [18,19]. While AMPK activation restores basal energy balance and preserves cell viability, it exerts a global suppressive effect on anabolic pathways. Specifically, activated AMPK directly inhibits the mechanistic target of rapamycin complex (mTORC) via the tuberous sclerosis complex 2 (TSC2) pathway, thereby blocking energy-intensive processes such as protein and lipid synthesis while simultaneously accelerating catabolism to generate ATP [20]. Consequently, sustained AMPK activation impairs the liver’s capacity to supply nutrients to peripheral tissues; instead, it drives excessive consumption of systemic metabolic resources, directly limiting the substrate reserves available to support muscle growth.

Over the past decade, extensive transcriptomic studies on grass carp have yielded substantial insights into its growth mechanisms. However, the transcriptional levels of genes (i.e., mRNA abundance) do not always correlate proportionally with the final protein expression levels [6,7,21]. The translation of mRNA into functional proteins is frequently modulated by complex post-transcriptional regulatory processes, including alterations in translation efficiency, protein degradation, alternative splicing, mRNA decay, and variations in translational efficiency [21,22]. Thus, sole reliance on transcriptomic data fails to accurately reflect the actual concentration of functional proteins in organisms. In the present study, we employed an integrated analytical strategy combining transcriptomics and data-independent acquisition (DIA)-based proteomics. This approach not only effectively mitigates “expression noise” inherent in single-omics analyses but also enables the dissection of expression correlations at both the transcriptional and protein levels in grass carp exhibiting differential growth.

In the present study, we integrated transcriptomics and data-independent acquisition (DIA)-based proteomics to perform multi-omics analyses at both the transcriptional and protein levels on the brain, liver, and muscle tissues of fast-growing (FG) and slow-growing (SG) grass carp reared under identical pond culture conditions. From a multi-organ perspective, we investigated the systemic decoupling phenomena of neuroendocrine regulation, muscle anabolism and liver energy metabolism behind the growth differences of grass carp under high-density farming conditions. Specifically, we aimed to clarify how the post-transcriptional activation of the mTOR pathway in the muscle of fast-growing individuals promotes the massive deposition of proteins, while the high-energy-consuming state driven by AMPK in the liver of slow-growing individuals restricts overall body growth. We hypothesize that growth differentiation is caused by an imbalance in energy allocation: in slow-growing fish, chronic stress and hepatic energy waste prevent the muscle from receiving the necessary substrates for protein synthesis, whereas fast-growing fish maintain an efficient metabolic state that favors muscle growth. Elucidating the crosstalk between muscle protein deposition and hepatic metabolic regulation not only deciphers the fundamental molecular mechanisms governing growth divergence in grass carp under high-density culture but also identifies key, high-confidence molecular targets for marker-assisted selection. Ultimately, this work will facilitate the development of superior grass carp strains optimized for modern high-density aquaculture systems, characterized by enhanced stress tolerance and rapid growth.

## 2. Materials and Methods

### 2.1. High-Density Experimental Design and Sampling

This experiment involving the high-density culture of grass carp (*Ctenopharyngodon idella*) was conducted over a 9-month period, from August 2024 to May 2025, at Guangdong Chengyi Industrial Group Co., Ltd. (Guangdong, China) in earthen ponds. On 5 August 2024, an initial population of 14,200 fish with an average body weight of 19 g was stocked. During the culture period, the fish were fed twice daily at a rate of 2% of their body weight, with the total feed allotment adjusted monthly based on the recalibrated total biomass. Throughout the trial, stocking density and dissolved oxygen (DO) levels were strictly monitored. In late September 2024, the stocking density reached a peak of 32,430 kg/ha (2162 kg/mu), with DO levels ranging from 1 to 5 mg/L. In contrast, typical farming densities during this period range from 13,110 to 16,605 kg/ha, with DO levels maintained between 3 and 7 mg/L. Consequently, the peak stocking density in this study was approximately 2.0 to 2.5 times higher than standard commercial densities. During the husbandry period, the stocking density was adjusted by periodically reducing the number of fish to maintain viable growth conditions. This high-intensity environment was sustained for 9 months to induce chronic physiological adaptations. At the conclusion of the trial in May 2025, the final population was approximately 9400 fish. Following the 9-month experimental period, an extreme phenotype sampling strategy was employed. A total of 25 fish representing the extremes of growth divergence—10 fast-growing (FG) and 15 slow-growing (SG) individuals—were purposively collected based on their significant differences in body weight and length. Morphological parameters, including body weight (g) and total length (cm), were measured for each selected individual using an electronic balance (accuracy ±0.01 g) and a standardized measuring board (accuracy ±0.1 cm), respectively. Following a standard 24-h fasting period, the fish were deeply anesthetized with MS-222 (100 mg/L) prior to tissue sampling. For subsequent multi-omics and validation analyses, a strict sampling strategy was applied to yield 4 biological replicates per cohort (*n* = 4): 4 individuals were randomly selected from the FG group as independent replicates, while 8 individuals from the SG group were selected and pooled pairwise (two individuals merged into one sample) to generate the 4 biological replicates. The brain, liver, and muscle tissues were then rapidly dissected, snap-frozen in liquid nitrogen, and stored at −80 °C for subsequent sequencing and molecular analyses. All animal procedures were approved by the Animal Care and Use Committee of the South China Sea Fisheries Research Institute, Chinese Academy of Fishery Sciences (No. SCSFRI96-253), and adhered to institutional ethical standards.

### 2.2. Transcriptome Sequencing and Assembly

Total RNA was extracted from brain, liver, and muscle tissues using Trizol reagent (Life Technologies, Shanghai, China) following the manufacturer’s protocol. To eliminate genomic DNA contamination, all RNA samples were treated with DNase I. RNA quality was validated by NanoDrop 2000 and Agilent 2100 Bioanalyzer (Agilent Technologies, Santa Clara, CA, USA) (RIN > 7.0). A total of 24 RNA samples (4 biological replicates per cohort) were utilized. In the fast-growing (FG) group, 4 individuals were selected as independent replicates; in the slow-growing (SG) group, 8 individuals were pooled pairwise to generate 4 biological replicates. Strand-specific mRNA libraries were constructed using the Hieff NGS^®^ Ultima Dual-mode mRNA Library Prep Kit (Yeasen, Shanghai, China) and sequenced on the DNBSEQ-T7 platform (MGI/BGI).

Raw sequencing data were filtered using fastp and Bowtie2 to remove low-quality reads and rRNAs. Clean reads were aligned to the Ctenopharyngodon idella reference genome using HISAT2 and assembled with StringTie. Gene expression levels were quantified using RSEM and normalized as TPM. The software and tools used in this study were cited as follows: fastp [23], Bowtie2 [24], HISAT2 [25], StringTie [26], RSEM [27].

### 2.3. Proteome Sequencing and Quantification

To ensure comprehensive multi-omics integration, 24 protein samples were prepared from the same tissues used for transcriptomic analysis. Protein samples were processed using the Micro Sample Pretreatment Kit (OSFP0001, Shanghai Omicsolution Co., Ltd., Shanghai, China), which involved protein denaturation, reduction, alkylation, and tryptic digestion. Digested peptides were desalted using a C18 desalting column and vacuum dried. The peptides were reconstituted in Phase A (0.1% formic acid in water) and analyzed via LC-MS/MS. Separation was performed on an AUR3-1575C18 analytical column (IonOpticks, Melbourne, Australia) using a 15-min gradient on a Vanquish Neo UHPLC system coupled to a timsTOF HT mass spectrometer (Bruker Daltonik, Bremen, Germany). The gradient was operated at a flow rate of 400 nL/min, with Phase B consisting of 80% acetonitrile with 0.1% formic acid. Data-independent acquisition (DIA) was performed in diaPASEF mode. The mass spectrometer was configured with a scan range of 380.8–1170.8 *m*/*z* and an isolation window width set to 10 Da. During the PASEF MS/MS scans, the collision energy was increased linearly with ion mobility, rising from 20 eV (1/K0 = 0.6 Vs/cm^2^) to 60 eV (1/K0 = 1.6 Vs/cm^2^). Retention times were automatically calibrated using iRT peptides (Biognosys, Schlieren, Switzerland). Raw DIA data were processed using Spectronaut (v20, Biognosys, Schlieren, Switzerland) with default BGS Factory Settings. Protein identification and quantification were performed with a false discovery rate (FDR) set at 1.0% for precursors, peptides, and proteins. Protein group quantification was achieved using the MaxLFQ algorithm.

### 2.4. Identification of DEGs and DEPs

Differential expression analysis at both the transcriptional and translational levels was conducted based on stringent thresholds. For the transcriptome, differentially expressed genes (DEGs) were identified using DESeq2 [28], with significance defined as a false discovery rate (FDR) < 0.05 and |log2 (fold change)| > 1. For the proteome, differentially expressed proteins (DEPs) were screened using Student’s *t*-test with Benjamini–Hochberg adjustment [29]. Proteins meeting the criteria of a fold change > 1.5 and a corrected *q*-value < 0.05 were defined as significant DEPs.

### 2.5. Functional Enrichment Analysis

Gene Ontology (GO) [30] and Kyoto Encyclopedia of Genes and Genomes (KEGG) [31] enrichment analyses for DEGs and DEPs were performed using hypergeometric tests. *p*-values were corrected using the Bonferroni (GO) and Benjamini–Hochberg (KEGG) methods, with a corrected *p* ≤ 0.05 as the significance threshold. Additionally, GSEA was conducted on the muscle proteome using GSEApy (v1.2.1) [32], which implements the algorithm originally described by Subramanian et al. [33]. Pathways with a nominal *p* < 0.05 and an FDR *q* < 0.25 were considered significantly enriched.

### 2.6. Validation of Omics Data

To validate the reliability of the multi-omics data, quantitative real-time PCR (qPCR) and parallel reaction monitoring (PRM) were employed. For both qPCR and PRM analyses, tissues from 6 selected individuals—3 from the FG group and 3 from the SG group—were utilized to provide 3 independent biological replicates (*n* = 3) per group. For transcriptome validation, cDNA was synthesized using SweScript All-in-One RT SuperMix (Wuhan, China), and qPCR was performed on a CFX Connect System (Hercules, CA, USA) using SYBR Green. Relative expression was calculated via the $2^{-△△CT}$ method [34], normalized to β-actin and rpl13a as internal references [35,36]. The specific primer sequences were designed for qPCR validation (Table S2). For proteome validation, 19 representative DEPs (8 from muscle, 11 from liver) were selected. Peptides prepared via the FASP method were spiked with iRT standards and analyzed on a Tims TOF HT mass spectrometer in prm-PASEF mode (Bruker Daltonics GmbH & Co. KG, Bremen, Germany). PRM data were robustly quantified and manually curated using Skyline software (v25.1).

### 2.7. Integrated and Statistical Analyses

Transcriptome-proteome correlation was evaluated by visualizing the overlap of DEGs and DEPs using Venn diagrams. Expression trends across the two omics layers were comprehensively assessed through nine-quadrant scatter plots. All routine statistical comparisons between groups were performed using SPSS 27.0. Data normality and variance homogeneity were pre-evaluated using the Shapiro–Wilk test and Levene’s test, respectively. For normally distributed data with homogeneous variances, Student’s *t*-test was employed; for data exhibiting unequal variances, Welch’s *t*-test was applied. Data are presented as mean ± standard deviation (SD), and a *p*-value ≤ 0.05 was considered statistically significant.

## 3. Results

### 3.1. Morphological Measurement

**Figure 1:**
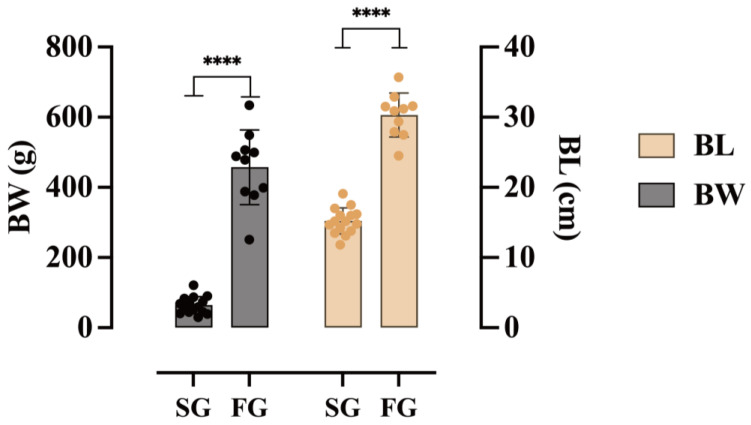
Morphological characteristics of fast- and slow-growing grass carp. Comparison of body weight (BW) and body length (BL) between fast-growing grass carp (FG, *n* = 10) and slow-growing grass carp (SG, *n* = 15). **** was significantly different (*p* < 0.0001).

In total, 25 grass carp were collected, including 15 slow-growing grass carp (SG) and 10 fast-growing grass carp (FG). The growth traits of these fish samples were measured, such as body weight and body length (Figure 1). The average values of body weight and body length from FG and SG were 457.31 and 64.27 g, 30.31 and 15.21 cm, respectively. Similarly, the values of body weight and body length from SG were both significantly smaller than that from FG (*p* < 0.0001). 

### 3.2. Transcriptome Analysis Between the FG and SG Groups

#### 3.2.1. Expression Profiles of mRNAs in Fast-Growing and Slow-Growing Fish

Transcriptome sequencing was subsequently performed on these three tissues. The strand-specific RNA-seq libraries yielded totals of 300.65 Gb of data. From these libraries, a total of 24,707 mRNAs were identified. All identified RNAs were successfully mapped to the grass carp *(Ctenopharyngodon idella*) genome. A high percentage (94–97.47%) of the clean reads successfully aligned to the reference genome, covering 25,210 annotated genes. Of these alignments, 79.78–91.06% were uniquely mapped (Table S1). The mRNA read count matrix and normalized expression levels (TPM) across the brain, liver, and muscle tissues are detailed in Table S4.

#### 3.2.2. Differential Transcriptomic Analysis of Muscle

**Figure 2:**
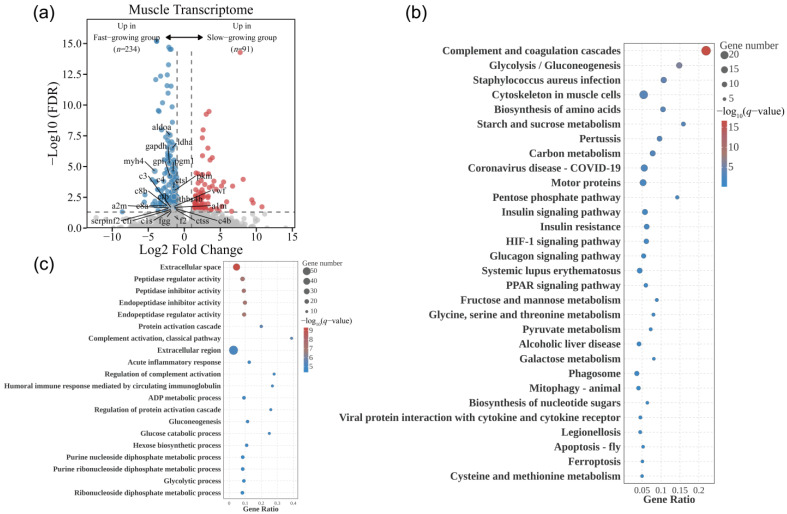
Transcriptome analysis of muscle tissue between fast- and slow-growing grass carp. (**a**) Volcano plot of differentially expressed genes (DEGs) in the muscle transcriptome (|log2 Fold Change| > 1, FDR < 0.05). Red dots represent genes significantly up-regulated in the slow-growing (SG) group, blue dots represent genes significantly up-regulated in the fast-growing (FG) group, and grey dots represent non-significantly differentially expressed genes. (**b**) KEGG pathway enrichment of the muscle transcriptome; (**c**) GO functional enrichment of muscle differentially expressed transcriptome.

A total of 325 differentially expressed genes were identified between FG and SG, including 91 genes upregulated in SG and 234 genes up-regulated in FG (Figure 2a).

In the KEGG enrichment analysis of FG muscle, the most significantly enriched pathway was “Complement and coagulation cascades” (e.g., *c3*, *c1s*, *cfb*, *f2*, *fgg*, *c4b*, *c4*, *vwf*, *cfi*, *c8a*, and *c8b*), implying active extracellular and immune signaling in large individuals. Meanwhile, carbohydrate catabolism and energy generation were markedly accelerated in FG muscle to meet the metabolic demand of rapid biomass accumulation. The “Glycolysis/Gluconeogenesis” and “Pyruvate metabolism” pathways were enriched. Multiple core enzyme nodes were up-regulated in the FG group (e.g., *pkm*, *gapdh*, *aldoa*, *gpi*, *pgm1*, and *ldha*), further satisfying the high metabolic requirement of muscle hyperplasia. Moreover, the “Muscle cytoskeleton” pathway was significantly enriched (e.g., *myh4*), providing further evidence for muscle tissue expansion in the FG group (Figure 2b).

GO enrichment analysis further confirmed the high metabolism and expansion of FG muscle. In the cellular component category, “extracellular space” was a significantly enriched term. Key regulators of cell communication and matrix remodeling, such as *thbs4b*, *ctsl*, and *ctss*, were enriched in FG muscle. In the molecular function domain, “peptidase regulator activity” and “peptidase inhibitor activity” were significantly enriched in the FG group. The FG group highly expressed protease inhibitors (e.g., *serpinf2*, *a1m*, and *a2m*). This may protect muscle proteins, prolonging their half-life and increasing protein biomass. FG muscle also up-regulated both “glucose catabolic process” and “hexose biosynthetic process”, probably through high *gapdh* and *pkm* expression, which supports the energetic basis of the growth difference (Figure 2c). 

Overall, the muscle transcriptome differed mainly in transcripts related to immune-mediated extracellular matrix remodeling, accelerated glycolysis, peptidase inhibition, and cytoskeletal contractile structures. This provides transcript-level evidence for the rapid hypertrophy observed in fast-growing grass carp.

#### 3.2.3. Differential Transcriptomic Analysis of Liver

**Figure 3:**
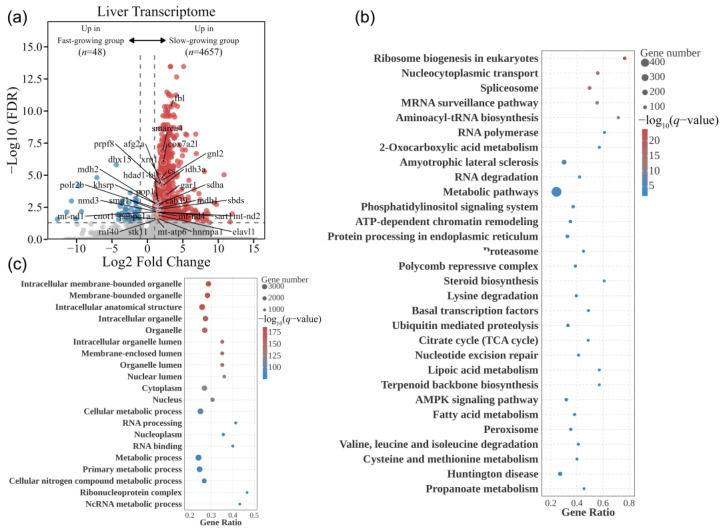
Transcriptome analysis of liver tissue. (**a**) Volcano plot of differentially expressed genes (DEGs) between fast- and slow-growing groups (|log2 FC| > 1, FDR < 0.05). Red dots represent genes significantly up-regulated in the slow-growing (SG) group, blue dots represent genes significantly up-regulated in the fast-growing (FG) group, and grey dots represent non-significantly differentially expressed genes. (**b**) KEGG pathway enrichment of the liver transcriptome; (**c**) GO functional enrichment of differentially expressed genes in the liver.

A total of 4705 differentially expressed genes were identified between FG and SG, including 4657 genes upregulated in SG and 48 genes up-regulated in FG (Figure 3a).

KEGG pathway enrichment analysis revealed significant activation of pathways related to genetic information processing and protein synthesis in the liver of the SG group. The “Ribosome biogenesis in eukaryotes” pathway was significantly enriched in the SG group (e.g., *afg2a*, *gar1*, *nmd3*, *sbds*, *xrn1*, *fbl*, *gnl2*, and *pop1*). Meanwhile, “Spliceosome” and “mRNA surveillance pathway” were also markedly activated (e.g., *smg1*, *rnf40*, *cnot1*, *prpf8*, and *sart1*). In addition, the “AMPK signaling pathway” was significantly enriched (e.g., *cab39*, *stk11*), indicating metabolic stress and an energy crisis. Correspondingly, the “Citrate cycle (TCA cycle)” and “Oxidative phosphorylation” pathways were activated, involving mitochondrial metabolic node genes such as *cs*, *mdh1*, *mdh2*, *idh3a*, and *sdha*, and respiratory chain components such as *mt-nd1*, *mt-nd2*, *mt-nd4*, *mt-atp6*, and *cox7a2l*. These results suggest a compensatory activation of aerobic respiration in the SG liver in response to energy stress. Thus, although ribosome biogenesis and mRNA processing activities were enhanced in slow-growing individuals, a concurrent pronounced energy stress may have limited efficient protein accumulation and growth (Figure 3b).

GO enrichment analysis further supported these findings. In the biological process (BP) category, “RNA processing” was the top enriched term, covering key processing factors specifically accumulated in the SG liver, such as dhx15. In the cellular component (CC) category, “nucleoplasm” was enriched with high-density structural and transcriptional response elements, including hdac1-b, polr2b, and smarca4, indicating significant nuclear structural reorganization in slow-growing individuals. In the molecular function (MF) category, the significant enrichment of “RNA binding” reflected a global acceleration of post-transcriptional modification systems, containing a wide range of structural and regulatory factors such as *hnrnpa1*, *elavl1*, *khsrp*, and *pabpc1a* (Figure 3c).

Overall, the liver transcriptome of slow-growing grass carp displayed an asymmetric remodeling pattern, characterized by marked up-regulation of ribosome biogenesis, spliceosome assembly, RNA processing, AMPK signaling, and mitochondrial energy catabolism. These results suggest increased metabolic activity that limits somatic growth potential.

#### 3.2.4. Differential Transcriptomic Analysis of Brain

**Figure 4:**
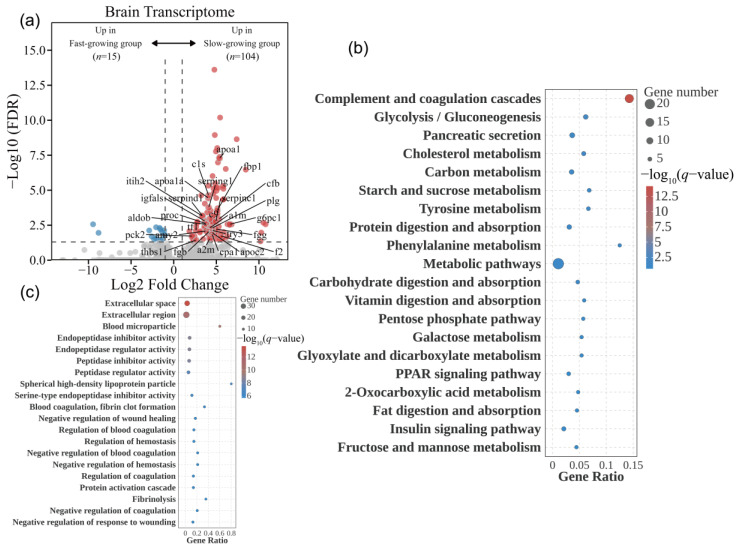
Transcriptome analysis of brain tissue. (**a**) Volcano plot of differentially expressed genes (DEGs) between fast- and slow-growing groups (|log2 FC| > 1, FDR < 0.05). Red dots represent genes significantly up-regulated in the slow-growing (SG) group, blue dots represent genes significantly up-regulated in the fast-growing (FG) group, and grey dots represent non-significantly differentially expressed genes. (**b**) KEGG pathway enrichment of the liver transcriptome; (**c**) GO functional enrichment of differentially expressed genes in the brain.

A total of 119 differentially expressed genes were identified between FG and SG, including 104 genes up-regulated in SG while 15 genes up-regulated in FG (Figure 4a).

KEGG pathway enrichment analysis revealed enrichment of pathways linked to central neuroinflammation and systemic stress responses in the SG brain. The “Complement and coagulation cascades” was the most significantly enriched pathway (including *a1m*, *a2m*, *c1s*, *proc*, *plg*, *cfb*, *fgg*, *c9*, *f2*, *serpind1*, *serping1*, and *fgb*). The “Glycolysis/Gluconeogenesis” pathway was also enriched, comprising key rate-limiting gluconeogenic enzymes (*pck2*, *fbp1*, *g6pc1*, *aldob*). This points to cellular energy deficiency and attempts at energy-costly de novo glucose synthesis in the SG brain. Moreover, “Pancreatic secretion” and “Carbohydrate digestion and absorption” pathways were ectopically activated in the brain (e.g., the traditional peripheral digestive enzymes *try3*, *amy2*, and *cpa1*), indicating aberrant central neural metabolism. The specific enrichment of “Cholesterol metabolism” and “PPAR signaling pathway” (e.g., *apoa1*, *apoa1a*, and *pck2*) further pointed to alterations in central lipid processing and hormone-related sensory networks (Figure 4b).

GO enrichment analysis further supported the neural abnormalities and central metabolic remodeling described above. In the biological process (BP) category, “blood coagulation, fibrin clot formation” and “negative regulation of wound healing” were the most significantly enriched functional terms. This enrichment was primarily driven by the coordinated up-regulation of core signaling nodes such as *fgg*, *f2*, *fgb*, *serpinc1*, and *serping1* in the SG brain. In the cellular component (CC) category, “extracellular space” and “extracellular region” were the main enriched terms. They contained a high density of stress-responsive and transport proteins, including *aldob*, *apoa1*, *apoc2*, *tf1*, *thbs1*, *igfals*, and *itih2*. These proteins specifically accumulated in slow-growing individuals (Figure 4c).

Overall, the brain transcriptome of the SG grass carp differed mainly in excessive complement cascade activation, initiation of central gluconeogenesis, ectopic digestive enzyme expression, and extracellular stress signaling. This implies that the organism may constrain somatic growth through central neural regulation.

### 3.3. Proteome Analysis Between the FG and SG Groups

#### 3.3.1. Overall Statistics for Proteome Sequencing

Proteomic analysis identified 25,845 precursors, 23,963 peptides, 2670 protein groups, and 2922 proteins in muscle, 51,007 precursors, 49,139 peptides, 5985 protein groups, and 6315 proteins in liver, and 81,152 precursors, 76,186 peptides, 7812 protein groups, and 8142 proteins in brain (Figure S1a). Most proteins were supported by 11 or more peptides (Figure S1b). PCA analyses showed high protein overlap within tissues and clear separation between FG and SG groups, supporting the reliability of the proteomic dataset for differential analysis (Figure S1c–e). To identify DEPs between fast-growing and slow-growing groups, at least a 1.2-fold change (FC) in protein expression level and *p* < 0.05 were set as criteria in which positive log2FC indicates higher abundance in SG, whereas negative log2FC indicates higher abundance in FG. The protein quantification matrix and corresponding differential expression levels across the three tissues are provided in Table S5.

#### 3.3.2. Differential Proteome Analysis of Muscle

**Figure 5:**
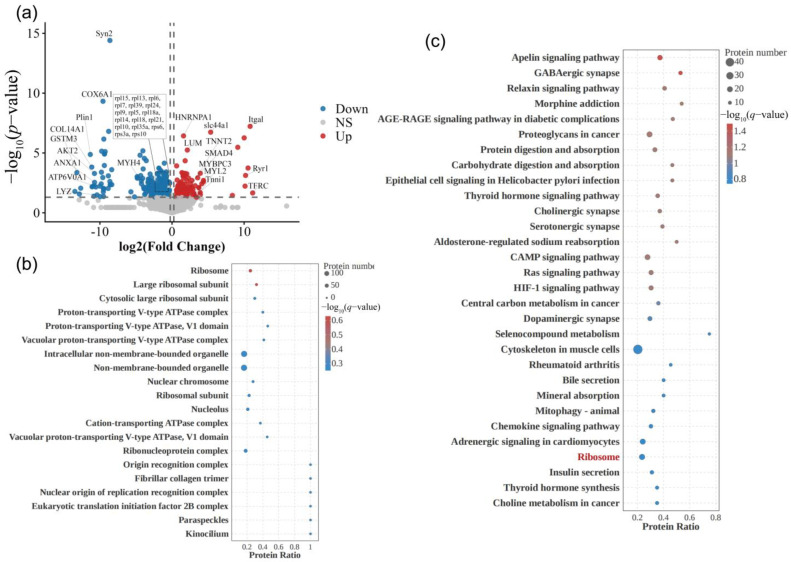
Differential proteome analysis of muscle tissue between fast- and slow-growing grass carp. (**a**) Volcano plot of differentially expressed proteins (DEPs) in muscle. Red dots in the upper right quadrant represent proteins with significantly higher expression levels in fast-growing groups, while blue dots in the upper left quadrant represent proteins with significantly lower expression levels (fold change ≥ 1.2 and *p* < 0.05); (**b**) GO functional enrichment of differentially expressed proteins in muscle; (**c**) KEGG pathway enrichment of differentially expressed proteins in muscle.

A total of 425 differentially abundant proteins were identified between FG and SG, including 167 proteins with higher abundance in SG and 258 proteins with higher abundance in FG. Proteins enriched in SG included Syn2, ITGAL, SLC44A1, HNRNPA1, LUM, TFRC, RYR1, MYL2, MYBPC3, TNNI1, MYH4, TNNT2, and SMAD4, which were mainly related to muscle structure, energy metabolism, membrane transport, immune response, and intracellular signaling. In contrast, proteins such as COX6A1, GSTM3, PLIN1, COL14A1, ATP6V0A1, AKT2, ANXA1, LYZ, and multiple RPL/RPS ribosomal proteins showed higher abundance in FG, indicating differences in energy metabolism, antioxidant response, lipid metabolism, ribosomal function, and immune-related processes (Figure 5a). GO enrichment analysis showed that the differentially abundant proteins were mainly enriched in ribosome, large ribosomal subunit, cytosolic large ribosomal subunit, ribosomal subunit, and ribonucleoprotein complex, suggesting marked differences in protein synthesis-related machinery between the two groups. Additional enriched terms, including proton-transporting V-type ATPase complex, V-type ATPase V1 domain, nuclear chromosome, nucleolus, origin recognition complex, and fibrillar collagen trimer, indicated that ion transport, chromatin-associated regulation, and extracellular structural organization were also altered in muscle tissue (Figure 5b). KEGG enrichment analysis further showed that the differentially abundant proteins were involved in the cytoskeleton in muscle cells, ribosome, protein digestion and absorption, apelin signaling pathway, cAMP signaling pathway, Ras signaling pathway, HIF-1 signaling pathway, aldosterone-regulated sodium reabsorption, and several neurotransmitter-related pathways, including GABAergic, cholinergic, serotonergic, and dopaminergic synapse. Among these pathways, cytoskeleton in muscle cells contained the largest number of mapped proteins, whereas the apelin signaling pathway, GABAergic synapse, and relaxin signaling pathway showed relatively stronger enrichment signals (Figure 5c). In total, the muscle proteome differed mainly in proteins related to contractile structure, ribosomal activity, extracellular matrix remodeling, ion transport, energy metabolism, and stress-responsive signaling.

#### 3.3.3. Differential Proteome Analysis of Liver

**Figure 6:**
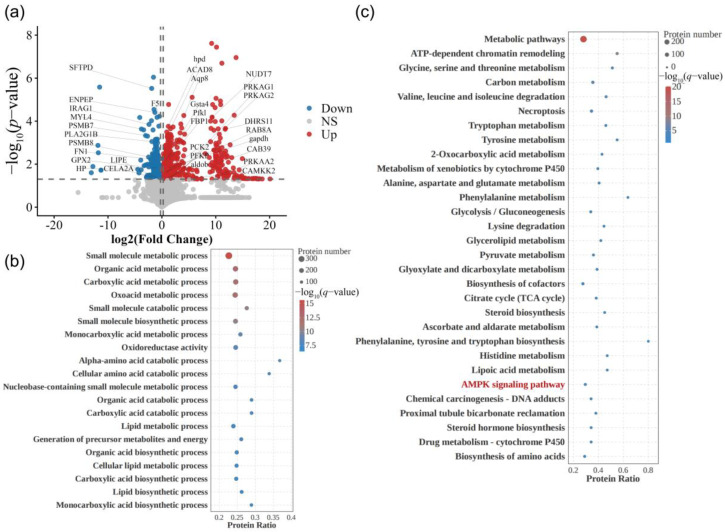
Differential proteome analysis of liver tissue between fast- and slow-growing grass carp. (**a**) Volcano plot of differentially expressed proteins (DEPs) in liver. Red dots in the upper right quadrant represent proteins with significantly higher expression levels in fast-growing groups, while blue dots in the upper left quadrant represent proteins with significantly lower expression levels (fold change ≥ 1.2 and *p* < 0.05); (**b**) GO functional enrichment of differentially expressed proteins in liver; (**c**) KEGG pathway enrichment of differentially expressed proteins in liver.

A total of 967 differentially abundant proteins were identified in liver tissue between FG and SG carp, including 750 proteins with higher abundance in SG and 217 proteins with higher abundance in FG. The SG-enriched proteins included several enzymes involved in carbohydrate, amino acid, lipid metabolism, energy sensing, glycolysis, and vesicle trafficking, such as ALDOB, GAPDH, FBP1, HPD, ACAD8, NUDT7, DHRS11, GSTA4, AQP8, SLC38A3, PCK2, CAMKK2, CAB39, PRKAA2, PRKAG1, PRKAG2, PFKP, PFKL, and RAB8A. In contrast, proteins showing higher abundance in FG included HP, CELA2A, PLA2G1B, GPX2, PSMB7, PSMB8, FN1, SFTPD, ENPEP, F5, IRAG1, MYL4, and LIPE which were mainly related to digestion, antioxidant response, proteasomal activity, extracellular structure, immune regulation, vascular-associated processes and lipid mobilization (Figure 6a). GO enrichment analysis showed that the liver differential proteome was strongly associated with the small molecule metabolic process, organic acid metabolic process, carboxylic acid metabolic process, oxoacid metabolic process, lipid metabolic process, amino acid catabolic process, oxidoreductase activity, and generation of precursor metabolites and energy (Figure 6b). These terms indicate that metabolic turnover, redox regulation and energy production were the major biological differences between the two groups (Figure 6b). KEGG enrichment analysis further confirmed a metabolism-dominated pattern. The differentially abundant proteins were mainly enriched in metabolic pathways, carbon metabolism, glycolysis/gluconeogenesis, pyruvate metabolism, citrate cycle, glycine/serine/threonine metabolism, valine/leucine/isoleucine degradation, tryptophan metabolism, tyrosine metabolism, phenylalanine metabolism, glycerolipid metabolism, steroid biosynthesis, steroid hormone biosynthesis, metabolism of xenobiotics by cytochrome P450, and drug metabolism–cytochrome P450. In addition, the AMPK signaling pathway and ATP-dependent chromatin remodeling were also enriched, suggesting that energy sensing and transcriptional regulation may be involved in liver metabolic divergence. In total, the liver proteomic differences between FG and SG group grass carp were mainly characterized by enhanced metabolic enzyme abundance in SG, especially proteins linked to central carbon metabolism, amino acid turnover, lipid metabolism, redox balance and detoxification-related pathways.

#### 3.3.4. Differential Proteome Analysis of Brain

**Figure 7:**
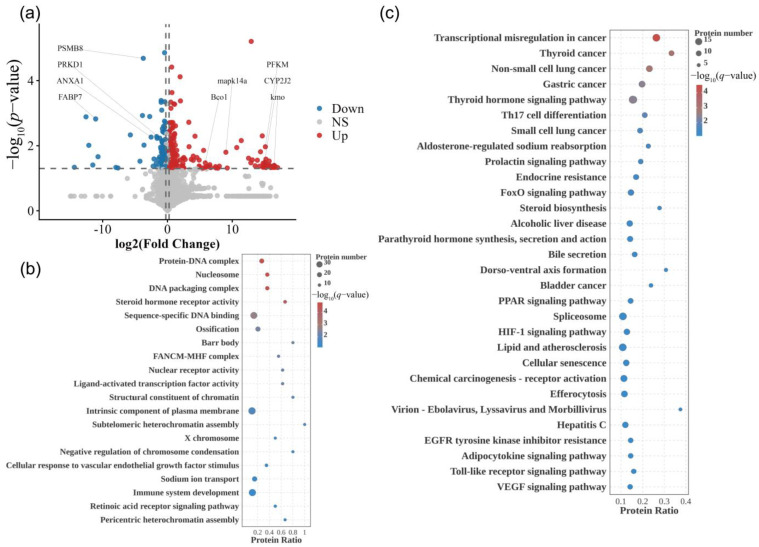
Differential proteome analysis of brain tissue between fast- and slow-growing grass carp. (**a**) Volcano plot of differentially expressed proteins (DEPs) in brain. Red dots in the upper right quadrant represent proteins with significantly higher expression levels in fast-growing groups, while blue dots in the upper left quadrant represent proteins with significantly lower expression levels (fold change ≥ 1.2 and *p* < 0.05); (**b**) GO functional enrichment of differentially expressed proteins in brain; (**c**) KEGG pathway enrichment of differentially expressed proteins in brain.

A total of 454 differentially abundant proteins were identified between the brain tissues of FG and SG groups of grass carp, including 306 proteins with higher abundance in SG and 148 proteins with higher abundance in FG. Representative proteins increased in SG included KMO, PFKM, CYP2J2, BCO1, RXR isoforms, and MAPK14A, whereas FABP7, PSMB8, ANXA1, transferrin-a, and PRKD1 showed higher abundance in FG. These proteins were mainly associated with metabolism, nuclear receptor signaling, immune regulation, ion transport, and intracellular signal transduction (Figure 7a). GO enrichment analysis showed that the differentially abundant proteins were mainly enriched in terms related to chromatin organization and transcriptional regulation, including protein–DNA complex, nucleosome, DNA packaging complex, sequence-specific DNA binding, structural constituent of chromatin, steroid hormone receptor activity, nuclear receptor activity, and ligand-activated transcription factor activity. Additional enriched terms, such as intrinsic component of plasma membrane, sodium ion transport, immune system development, cellular response to vascular endothelial growth factor stimulus, and heterochromatin assembly, indicated that membrane transport, immune-related processes, and vascular signaling also differed between the two groups (Figure 7b). KEGG enrichment analysis further revealed that these proteins were involved in several endocrine, metabolic, immune, and signaling pathways. The most enriched pathways included thyroid hormone signaling, Th17 cell differentiation, aldosterone-regulated sodium reabsorption, prolactin signaling, FoxO signaling, steroid biosynthesis, PPAR signaling, spliceosome, HIF-1 signaling, Toll-like receptor signaling, and VEGF signaling pathways. Several KEGG disease-reference pathways, such as transcriptional misregulation in cancer, thyroid cancer, non-small cell lung cancer, and gastric cancer, were also enriched, mainly reflecting shared regulatory modules involving MAPK/AKT/RXR-related signaling proteins rather than disease-specific processes (Figure 7c). Overall, the proteomic differences in brain tissue between FG and SG grass carp were mainly characterized by changes in chromatin-associated regulation, nuclear receptor-mediated transcription, endocrine signaling, lipid and energy metabolism, ion transport, and immune–vascular signaling.

### 3.4. Correlations Analysis Between Transcriptome and Proteome Data

#### 3.4.1. Integration Analysis of Transcriptomic and Proteomic Data in Muscle

**Figure 8:**
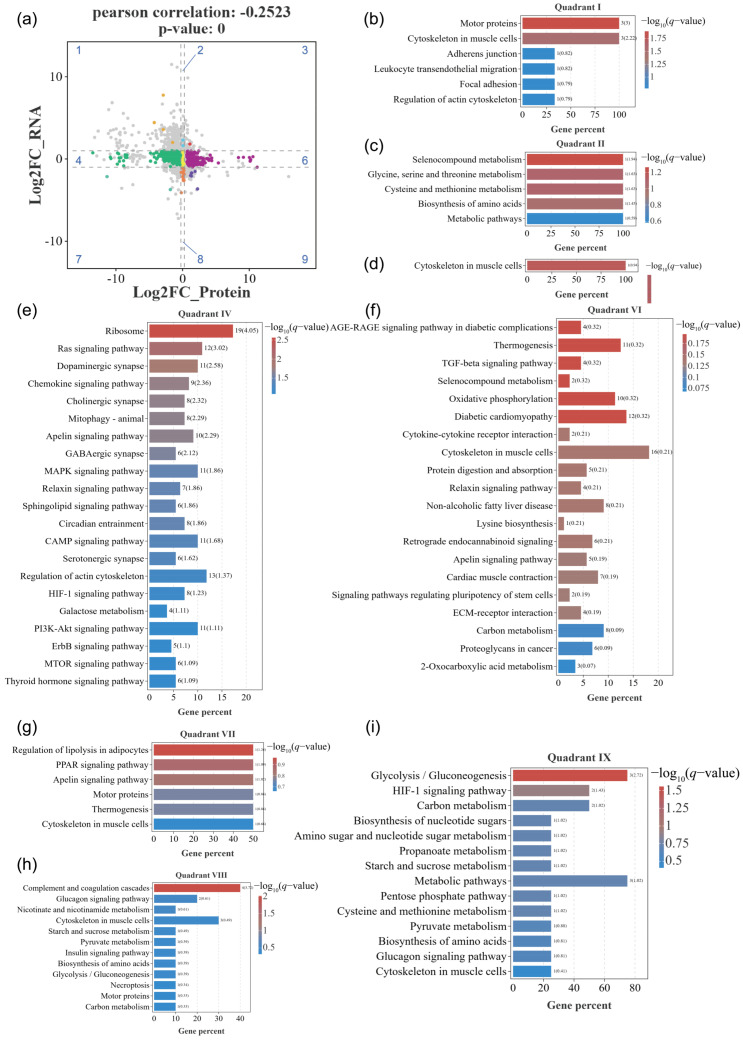
Integrated transcriptomic and proteomic analysis of grass carp skeletal muscle. (**a**) Nine-quadrant scatter plot illustrating the expression correlation between the transcriptome (Log2FC_RNA) and proteome (Log2FC_Protein) in fast- and slow-growing grass carp. Genes meeting strict dual-omics thresholds (|FC_RNA| ≥ 2, FDR ≤ 0.05; |FC_Protein| ≥ 1.2, *p* < 0.05) are highlighted in color. (**b**–**i**) KEGG pathway enrichment analysis of differentially expressed gene–protein pairs distributed in specific quadrants: (**b**) Quadrant I; (**c**) Quadrant II; (**d**) Quadrant III; (**e**) Quadrant IV (representing translationally enhanced networks in FG fish); (**f**) Quadrant VI (representing stress and energy dissipation in SG fish); (**g**) Quadrant VII; (**h**) Quadrant VIII and (**i**) Quadrant IX.

To systematically elucidate the regulatory mechanisms of grass carp skeletal muscle growth at a multi-omics level, we performed a deep integration of muscle transcriptomic and proteomic data. Through sequence mapping and co-expression analysis, a total of 2382 matched gene–protein pairs were successfully identified between the transcriptome and proteome. To precisely capture significantly differentially expressed molecules, stringent dual-omics thresholds were applied (RNA: |FC_RNA| ≥ 2 and FDR ≤ 0.05; Protein: |FC_Protein| ≥ 1.2 and *p*-value ≤ 0.05). Within the nine-quadrant scatter plot (Figure 8a), 350 gene–protein pairs met these criteria and were highlighted in significant colors. These significant pairs were mapped to the KEGG Orthology (KO) database to serve as subsequent enrichment analysis. The data distribution revealed that, in addition to the co-expressed genes along the diagonal, a substantial number of genes were distributed in quadrants exhibiting expression decoupling. Notably, while the standalone transcriptomic analysis failed to enrich for master growth-regulatory pathways (such as mTOR), this integrated analysis successfully captured these hidden signals, strongly suggesting that post-transcriptional regulation plays a decisive role in the growth divergence of grass carp skeletal muscle.

Quadrant IV (Figure 8e) of the nine-quadrant plot represents a gene set characterized by significantly up-regulated protein expression in FG individuals (FG) despite unchanged transcript (RNA) levels. This expression pattern is generally considered a hallmark of the release of post-transcriptional translational inhibition or an enhancement in translation efficiency. Strikingly, KEGG enrichment analysis of this quadrant successfully captured the complete mTOR signaling pathway and its core upstream and downstream cascades, including the Apelin, PI3K-Akt, and MAPK signaling pathways. It is worth noting that standalone proteomic KEGG analysis failed to enrich these pathways effectively, as the subtle fluctuations in signal kinase abundance often fall below conventional global thresholds. In contrast, the nine-quadrant expression misalignment analysis precisely amplified these subtle yet critical post-transcriptional signal shifts. To further evaluate the coordination status of this anabolic cascade at the global proteomic level, we performed Gene Set Enrichment Analysis (GSEA). The results revealed a highly significant overall upward synergistic shift of the Ribosome (NES = −2.259, *p*-Value < 0.01), mTOR signaling pathway (NES = −1.852, *p*-value < 0.05), and PI3K-Akt signaling pathway (NES = −1.748, *p*-Value < 0.05) in the FG group muscle proteome (Figure S2, Table S3). Analysis of the core genes extracted by GSEA revealed that key molecules—including kinase hubs (AKT2, MAPK1), critical downstream translation initiation factors (eIF4E), the extracellular growth factor scaffold (IGFALS), and the target ribosomal protein (RPS6)—underwent significant post-transcriptional accumulation in the muscle of FG fish. These findings strongly suggest that FG individuals specifically “license” the protein translation of growth-promoting networks, thereby triggering robust muscle ribosome assembly and biogenesis. To visually integrate this intricate post-transcriptional network, a comprehensive cascade was mapped based on the GSEA core enrichment leaders. Intriguingly, the orchestrating determinants spanned across multiple layers of upstream inputs and local physiological cues. Beyond the primary somatotropic kinase hubs (*akt2*, *mapk1*), a cascade of key intracellular mediators (e.g., *rras*, *rras2*, *rac1*) and structural integrators (e.g., *itga7*, *itgb4*) demonstrated consistent post-transcriptional stabilization or protein-level enrichment in the FG group. Moreover, a sub-cluster of vacuolar H+-ATPase subunits (*atp6v1a*, *atp6v1b2*, *atp6v1h*, *atp6v1c1a*) along with the amino acid transporter partner *slc3a2* exhibited coordinated post-transcriptional licensing, collectively providing data-driven verification for the systemic and localized hyperactivation of the muscle growth command.

Quadrant VI (Figure 8f) represents a gene set characterized by aberrantly up-regulated protein expression in individuals (SG) with no significant changes in transcript levels. KEGG enrichment analysis of this quadrant reveals the severe challenges present within the muscle microenvironment of the SG individuals. In the SG group, the TGF-beta signaling pathway exhibits significant post-transcriptional activation; this pathway (involving factors such as myostatin) serves as a classic negative regulatory network for teleost muscle growth. Concurrently, a substantial number of proteins associated with Thermogenesis, Oxidative phosphorylation, and the AGE-RAGE signaling pathway specifically accumulate in the SG group. The abnormal translation and accumulation of these proteins indicate that the skeletal muscle of the SG fish is trapped in a severe state of futile energy dissipation (energy leakage) and metabolic stress, thereby further exacerbating their growth stagnation phenotype.

Beyond the pronounced translational “licensing” and “stress” signatures observed in Quadrants IV and VI, the remaining quadrants (I, VIII, and IX) captured the complex temporal and buffering dynamics of skeletal muscle remodeling (Figure 8a,b,h). Although the number of genes in these quadrants was relatively small, they exhibited profound expression decoupling of structural components (“Motor proteins”, “Cytoskeleton in muscle cells”, e.g., MYH4, TNNI2, DES) and core metabolic enzymes (“Glycolysis/Gluconeogenesis”, e.g., GAPDH, LDHA, PKM). This complex bidirectional decoupling indicates that the abundance of massive myofibrillar proteins and highly expressed glycolytic machinery is subject to intensive post-transcriptional buffering to maintain structural and metabolic homeostasis. Furthermore, the concordant down-regulation observed in Quadrant VII (Figure 8g) highlighted the suppression of the “Regulation of lipolysis in adipocytes” pathway (featuring Plin1) in the FG group, indicating a concurrent shift in lipid droplet dynamics and substrate utilization accompanying muscle hypertrophy.

#### 3.4.2. Integration Analysis of Transcriptomic and Proteomic Data in Liver

**Figure 9:**
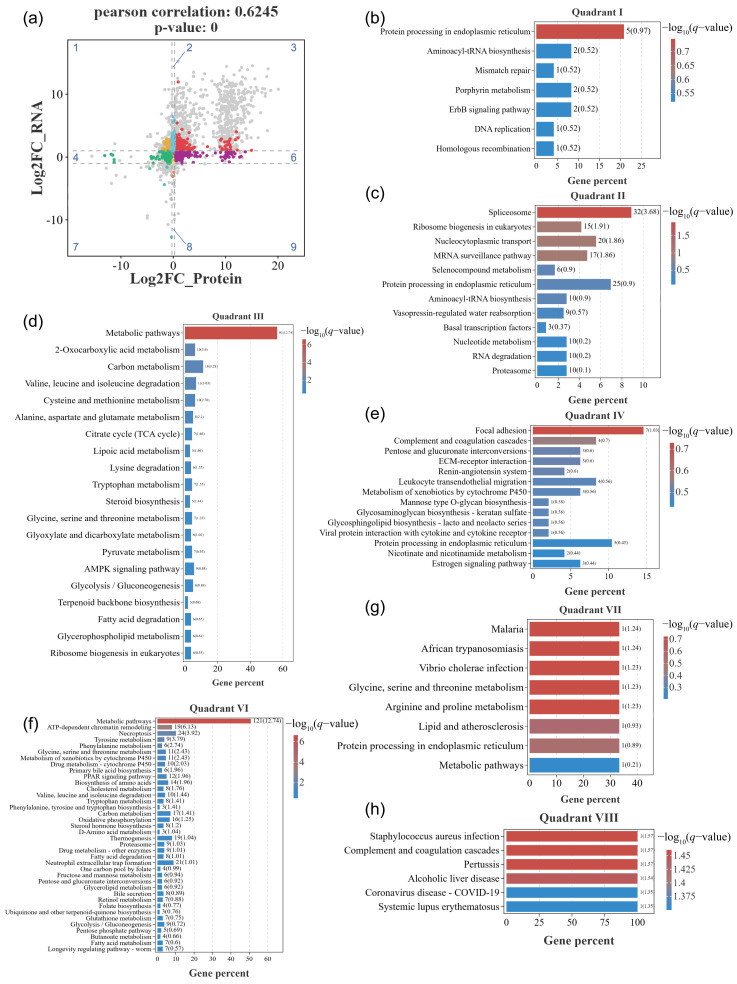
Integrated transcriptomic and proteomic analysis of grass carp liver. (**a**) Nine-quadrant scatter plot illustrating the expression correlation between the transcriptome (Log2FC_RNA) and proteome (Log2FC_Protein) in fast- and slow-growing grass carp. Numbers 1–9 indicate the nine quadrants, representing the nine distinct patterns of correlation between RNA and protein expression levels for the same gene. Genes meeting strict dual-omics thresholds (|FC_RNA| ≥ 2, FDR ≤ 0.05; |FC_Protein| ≥ 1.2, *p* < 0.05) are highlighted in color. (**b**–**h**) KEGG pathway enrichment analysis of differentially expressed gene–protein pairs distributed in specific quadrants: (**b**) Quadrant I and (**c**) Quadrant II (representing transcription–translation misalignment and translational bottlenecks in slow-growing fish); (**d**) Quadrant III; (**e**) Quadrant IV (representing enhanced post-transcriptional translation in fast-growing fish); (**f**) Quadrant VI (representing profound metabolic stress and energy expenditure in slow-growing fish); (**g**) Quadrant VII; and (**h**) Quadrant VIII.

To systematically elucidate the response mechanisms of grass carp liver metabolism to growth divergence, we performed a deep integration of liver transcriptomic and proteomic data. Through sequence alignment and quantitative mapping, a total of 5310 matched gene–protein pairs were identified. Applying stringent dual-omics thresholds (RNA: |FC_RNA| ≥ 2 and FDR ≤ 0.05; Protein: |FC_Protein| ≥ 1.2 and *p*-value ≤ 0.05), 1419 pairs exhibited significant differences (Figure 9a). These significant pairs were mapped to the KO database to serve as subsequent enrichment analysis.

The nine-quadrant scatter plot revealed a pronounced decoupling between the liver transcriptome and proteome. Molecules distributed in Quadrant III (227 pairs, concordant up-regulation of mRNA and protein in the SG group) and Quadrant VII (Figure 9f) (concordant up-regulation in the FG group) showed consistent expression trends. However, a substantial number of molecules were distributed in non-diagonal quadrants (such as Q1, Q2, Q4, and Q6), indicating that the metabolic homeostasis of the liver is governed by complex post-transcriptional regulation during the process of growth differentiation.

Enrichment analysis revealed distinct functional profiles across different regulatory quadrants. In Quadrant III (Q3), significant enrichment was observed in Metabolic pathways, Fatty acid metabolism, and Glycolysis/Gluconeogenesis, featuring key enzymes such as *fasn*, *g6pc1*, and *acss2*. Notably, Quadrant IV (Q4) (Figure 9d), characterized by stable mRNA levels but significantly up-regulated proteins in the FG group, reflected a specific enhancement of post-transcriptional translation. This quadrant was enriched in Focal adhesion, Complement and coagulation cascades, and Protein processing in endoplasmic reticulum; the specific accumulation of core proteins such as *vasp*, *pxn*, and *serping1* suggests that the FG liver possesses superior structural integrity and innate immune defense.

Conversely, the enrichment profile of Quadrant VI (Q6) (Figure 9e) (protein up-regulation in the SG group despite unchanged mRNA) underscored a state of profound metabolic stress in SG individuals. This quadrant was significantly enriched in Nonalcoholic fatty liver disease (NAFLD), Oxidative phosphorylation, and the AMPK signaling pathway. The elevated abundance of core proteins, including energy-sensing factors (*prkag1/2*), mitochondrial respiratory chain components (*ndufv1*, *cox4i1*), and critical gluconeogenic enzymes (*pck2*, *fbp1*), indicates that the SG liver is burdened by high energy expenditure and oxidative stress.

Furthermore, Quadrants I (Q1) and II (Q2) (Figure 9b,c) unveiled a severe “transcription–translation misalignment” in the SG group. Q1 (up-regulated mRNA but down-regulated protein) was significantly enriched in Protein processing in endoplasmic reticulum (e.g., *hspa5*, *pdia6*) and Aminoacyl-tRNA biosynthesis, while Q2 (up-regulated mRNA but unchanged protein) concentrated on the Spliceosome and Basal transcription factors. These patterns collectively suggest that while the SG group aggressively attempts to drive the transcription of protein synthesis-related genes, it encounters a critical translational bottleneck.

#### 3.4.3. Integration Analysis of Transcriptomic and Proteomic Data in Brain

**Figure 10:**
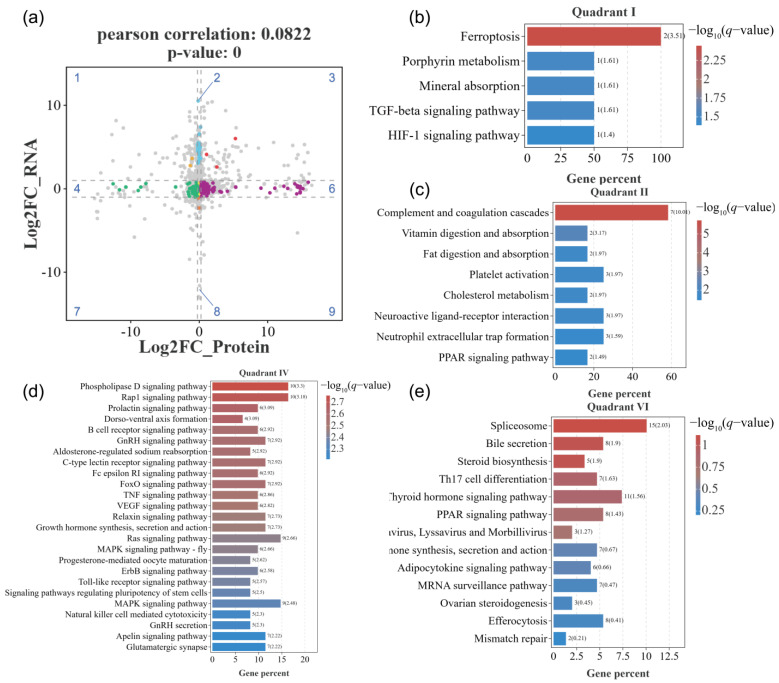
Integrated transcriptomic and proteomic analysis of grass carp brain. (**a**) Nine-quadrant scatter plot illustrating the expression correlation between the transcriptome (Log2FC_RNA) and proteome (Log2FC_Protein) in fast- and slow-growing grass carp. Numbers 1–9 indicate the nine quadrants, representing the nine distinct patterns of correlation between RNA and protein expression levels for the same gene. Genes meeting strict dual-omics thresholds (|FC_RNA| ≥ 2, FDR ≤ 0.05; |FC_Protein| ≥ 1.2, *p* < 0.05) are highlighted in color. (**b**–**e**) KEGG pathway enrichment analysis of differentially expressed gene–protein pairs distributed in specific quadrants: (**b**) Quadrant I and (**c**) Quadrant II; (**d**) Quadrant IV; and (**e**) Quadrant VI.

To systematically elucidate the central neuroendocrine and metabolic response mechanisms driving growth divergence, an integrated analysis of the grass carp brain transcriptome and proteome was conducted. Through quantitative mapping, a total of 2625 co-expressed gene–protein pairs were identified. To precisely capture biologically significant regulatory decoupling, stringent dual-omics thresholds were applied (RNA: |FC_RNA| ≥ 2 and FDR ≤ 0.05; Protein: |FC_Protein| ≥ 1.2 and *p*-value ≤ 0.05). Based on these criteria, 458 significant gene–protein pairs were distributed across a nine-quadrant scatter plot (Figure 10a). These significant pairs were mapped to the KO database to serve as subsequent enrichment analysis. The distribution revealed a profound transcription–translation misalignment, particularly within the SG group.

Quadrants I (Q1) and II (Q2) represented gene sets characterized by robust transcriptional up-regulation in the SG brain, which completely failed to translate into increased protein abundance. Specifically, Q1 (Figure 10b) (up-regulated mRNA but down-regulated protein in SG) was significantly enriched in stress-responsive pathways, including Ferroptosis, Porphyrin metabolism, and the HIF-1 and TGF-beta signaling pathways. Similarly, Q2 (Figure 10c) (up-regulated mRNA but unchanged protein in SG) was strongly enriched in Complement and coagulation cascades (e.g., *f2*, *fgg*, *fgb*) and Cholesterol metabolism. This extensive accumulation of transcripts without corresponding protein products indicates that the SG brain experiences reduced protein synthesis under chronic stress.

This translational bottleneck in the SG brain was further corroborated by the specific enrichment profile of Quadrant VI (Q6) (Figure 10e), representing proteins accumulated in the SG group despite unchanged mRNA levels. This quadrant was overwhelmingly enriched in RNA processing machineries, notably the Spliceosome and mRNA surveillance pathways. The post-transcriptional accumulation of numerous spliceosomal core components (such as *rbm8a*, *cdc5l*, and *ddx39b*) suggests that the SG brain is burdened by a severe transcription and RNA-splicing overload, forcing the accumulation of quality-control proteins to cope with transcription–translation uncoupling.

Conversely, Quadrant IV (Q4) (Figure 10d) highlighted the post-transcriptional translational advantage of the fast-growing (FG) group, featuring stable mRNA levels but significantly up-regulated proteins. KEGG enrichment successfully captured classic pro-growth and neuroplasticity networks, including the MAPK signaling pathway, Rap1 signaling pathway, and Phospholipase D signaling pathway. The specific accumulation of core kinase hubs, such as *map2k1*, *mapk1*, *hras*, and *akt3*, demonstrates that the FG individuals possess a highly efficient post-transcriptional “license” in the central nervous system, maintaining robust neuroendocrine signaling without excessive energy-consuming transcriptional bursts.

### 3.5. Validation of the Transcriptome and Proteome Data

**Figure 11:**
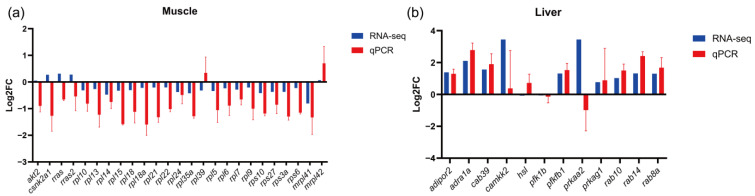
Validation of transcriptomic data by quantitative real-time PCR (qRT-PCR). Comparison of the log2 (Fold Change) values obtained from RNA-seq and qRT-PCR for selected differentially expressed genes (DEGs). (**a**) Muscle-related genes. (**b**) Liver-related genes. Data are presented as mean ± SD.

In total, 42 differentially expressed genes (DEGs) were selected for quantitative real-time PCR (qRT-PCR) validation to confirm the reliability of the RNA-seq results. These selected targets comprised muscle-related genes, including signaling hubs, general metabolic regulators (*akt2*, *csnk2a1*, *fbp2*, *mrpl41*, *mrpl42*, *pck2*, *ppp2r5e*, *rps10*, *rps27*, *rps3a*, *rps6*, *rras*, and *rras2*), and a substantial panel of ribosomal proteins *(rpl5*, *rpl6*, *rpl7*, *rpl9*, *rpl10*, *rpl13*, *rpl14*, *rpl15*, *rpl18*, *rpl18a*, *rpl21*, *rpl22*, *rpl24*, *rpl35a*, and *rpl39*) (Figure 11a). Concurrently, key liver-related metabolic and energy-sensing genes (*adipor2*, *adra1a*, *cab39*, *camkk2*, *hsl*, *pfk1*, *pfkfb1*, *prkaa2*, *prkag1*, *prkag2*, *rab10*, *rab14*, and *rab8a*) were also validated (Figure 11b). The expression patterns of these DEGs measured by qRT-PCR were generally consistent with the RNA-seq data, demonstrating the accuracy of the transcriptomic profiling. In addition, 19 DAPs were selected for PRM validation, including eight muscle proteins and eleven liver proteins. The PRM results supported the DIA trends for selected ribosome-related proteins in muscle and AMPK/metabolism-related proteins in liver, supporting the reliability of the proteomic results (Table 1). In general, the trends in the expression changes measured by PRM and DIA were consistent.

## 4. Discussion

### 4.1. Central Stress Response and Translational Arrest in the Brain of SG Individuals

In teleost fish, somatic growth is fundamentally dictated by the central nervous system (CNS), which acts as the supreme pacemaker integrating environmental cues, nutritional status, and neuroendocrine signaling [10]. Our multi-omics alignment revealed that the CNS of the slow-growing (SG) cohort is locked in a pathological state of severe chronic stress and neuroinflammation, paralyzing its capacity to emit growth-promoting cues. A key driver of this central dysfunction is the hyperactivation of the complement cascade (characterized by transcriptional surges of *fgg*, *f2*, *c1s*, and *a2m*) [37] and the p38 MAPK/KMO pathway. Chronic aquaculture stressors cause a pathological shunting of tryptophan metabolism toward the neurotoxic kynurenine pathway via KMO accumulation, deplete the precursor pool for serotonin synthesis, and mechanistically explain the disrupted appetite in SG fish [3,38].

Concurrently, the SG brain experiences a severe metabolic crisis, shifting energy toward energetically expensive de novo glucose synthesis (evidenced by PFKM accumulation) and de-sensitizing the CNS to systemic endocrine cues via aberrant expression of RXR isoforms [9,39]. This local energy deficit disrupts crucial central endocrine-sensing hubs, generating a false “starvation signal” that halts peripheral anabolic growth [40]. Notably, the ectopic central expression of traditional digestive enzyme genes (*try3*, *amy2*, *cpa1*) underscores a profound breakdown of neuro-metabolic circuitry regulating appetite homeostasis [41]. This “futile transcription” accompanied by spliceosome stress (*rbm8a*, *cdc5l*) indicates a severe translational bottleneck under chronic stress [21,42]. Conversely, the fast-growing (FG) brain exhibits peak neuroendocrine efficiency through efficient post-transcriptional “licensing” of pro-growth and neuroplasticity cascades (e.g., MAPK and Rap1 pathways) [43]. Supported by homeostatic chaperones like FABP7 and Annexin A1 (ANXA1), the FG brain actively buffers metabolic and inflammatory challenges to maintain its supreme neuroendocrine pacemaking capacity [44,45].

### 4.2. Hepatic AMPK-Mediated Metabolic Stress and Energy Futile Cycles

As the central hub of systemic metabolism, the liver dictates nutrient partitioning to support peripheral growth. In the SG liver, instead of allocating resources to growth, a massive transcriptional overdrive traps the tissue in an energy-draining “futile cycle” of hyperactive molecular turnover (notably within the spliceosome and proteolysis machinery) [46,47]. Crucially, skeletal muscle development relies on essential amino acids, particularly branched-chain amino acids (BCAAs), which serve as critical signaling molecules for translation [48]. Our data demonstrate that under chronic stress, the SG liver acts as a destructive “nutrient sink,” comprehensively upregulating BCAA degradation to fuel mitochondrial respiration rather than exporting these building blocks to the circulation [49,50].

This hepatic metabolic inefficiency is tightly governed by the central energy sensor AMPK. The robust enrichment of AMPK-related signaling components (including CAMKK2, CAB39, PRKAA2, and PRKAG1/2) together with mitochondrial respiratory chain complexes (*ndufv1*, *cox4i1*) indicates the hyperactivation of the hepatic AMPK-dependent energy-monitoring program [19,51]. Under chronic stress, this program accelerates ATP-generating catabolism, glycolysis, and mitochondrial oxidation to maintain basic cell viability at the expense of somatic anabolism [52,53]. This translational bottleneck and metabolic overdrive create a severe substrate starvation for downstream peripheral tissues [21,54]. In stark contrast, the FG liver exhibits profound transcriptional quiescence, reflecting optimal metabolic efficiency where dietary nutrients are seamlessly processed and reallocated to support skeletal muscle hypertrophy [17,47].

### 4.3. Post-Transcriptional Activation of mTOR Signaling and Ribosome Biogenesis Drives Muscle Hypertrophy

Skeletal muscle hypertrophy is an exceptionally energy-demanding process requiring precise coordination of nutrient availability and translational capacity [13]. In FG individuals, heightened glycolytic flux (evidenced by *gapdh* and *pkm*) and activated insulin signaling efficiently funnel metabolic intermediates toward rapid biomass accumulation [55]. This hyper-anabolic state is reinforced by a “translation-retention” strategy, where endogenous protease inhibitors (such as *a2m* and *serpins*) suppress protein degradation to maximize net myofibrillar accretion [56,57]. Furthermore, localized complement activation facilitates macrophage-mediated extracellular matrix (ECM) remodeling, a prerequisite for muscle satellite cell activation and fiber hypertrophy [58,59,60,61].

At the proteomic level, our integrated analysis resolved a critical regulatory paradox: while master growth-regulatory pathways remain transcriptionally silent, the mTORC1 signaling axis is robustly activated via post-transcriptional translational licensing [21,62]. Extracellular somatotropic priming, stabilized by the post-transcriptional accumulation of INS and the IGFALS complex, works in tandem with localized lysosomal nutrient sensing to unlock full mTORC1 activity [63]. This extracellular signal is flawlessly transduced downstream via core regulatory adaptors (GRB2, CRKL) and G-protein superfamily GTPases (including RRAS, RRAS2, and RAC1) to relieve the tonic repression on Rheb by the TSC2/YWHABA complex [64]. In parallel, this somatotropic activation works in synergy with a highly coordinated post-transcriptional stabilization of the vacuolar H+-ATPase complex (specifically its subcomponents *atp6v1a*, *atp6v1b2*, *atp6v1h*, and *atp6v1c1a*) and the amino acid transporter escort SLC3A2 [64,65]. Together, they form an indispensable inside-out apparatus that senses intracellular BCAAs and recruits mTORC1 directly to the lysosomal surface for full structural activation [65,66]. This dual neuroendocrine and nutrient-sensing network triggers terminal kinase execution (characterized by downstream accumulation of AKT2, MAPK1, EIF4E, and CSNK2A1) [67], driving robust ribosome biogenesis (via RPLs and RPSs up-regulation) and ribosome-driven translational intensity [62,68] to yield massive myofibrillar protein accretion (*myh4*, *tnnt2*) in the hyper-anabolic muscle microenvironment [55,61].

### 4.4. Multi-Organ Coordination of the Brain, Liver, and Muscle Drives Growth Divergence in Grass Carp

Our integrated multi-omics framework establishes a unified three-tissue coordination model for growth divergence in teleosts, where the brain acts as the primary pacemaker [8,11,69,70], the liver as the metabolic gatekeeper [71], and skeletal muscle as the terminal executor. Compared with previous growth-related omics studies in teleosts, such as single-tissue transcriptomic profiling of grass carp [2,3,4,5] or proteomic evaluations of growth enhancement in rainbow trout [6], our multi-organ integration reveals a shared global theme where metabolic allocation and translational machinery biogenesis dictate growth performance. Crucially, while previous single-omics frameworks primarily captured broad changes in transcript abundance [2,3,4,5], our integrated dataset uniquely demonstrates that core growth cascades are governed by intensive post-transcriptional buffering and licensing under aquaculture stress [21].

In fast-growing (FG) individuals, an efficient central neuroendocrine command minimizes central energy dissipation and transmits robust growth signals (buffered by neural FABP7 and ANXA1 protective networks). This allows the liver to operate in a state of peak metabolic efficiency and transcriptomic quiescence, acting as a seamless conduit that optimally reallocates essential amino acids and dietary nutrients to the peripheral circulation. This favorable systemic environment flawlessly licenses the muscle mTORC1 axis—driven by synergistic extracellular somatotropic priming (INS/IGFALS) and localized lysosomal BCAA sensing (v-ATPase)/SLC3A2—to accelerate ribosome biogenesis and drive massive myofibrillar protein accretion. Conversely, growth retardation in slow-growing (SG) fish is driven by systemic energetic collapse. Chronic aquaculture stress induces central neuroinflammation, pathological tryptophan-kynurenine shunting (KMO), and central translational arrest, emitting false starvation signals via aberrant RXR expression. Forced by these systemic disruptions, the liver activates an AMPK-mediated energy-monitoring program, trapping the tissue in energy-draining futile cycles that aggressively catabolize critical BCAAs for local hepatocyte survival. Deprived of both systemic anabolic drives and indispensable macromolecular building blocks, the downstream skeletal muscle remains trapped in translational dormancy, culminating in growth stagnation [21].

### 4.5. Study Limitations and Future Perspectives

Although this study offers a multi-organ, multi-omics landscape of grass carp growth divergence, certain limitations remain. First, all experimental fish were sourced from a single pond culture system, which restricts our ability to fully separate endogenous biological traits from micro-environmental confounding variables. Second, while our transcriptomic–proteomic alignment provides high-confidence biological insights, the identified post-transcriptional regulatory hubs remain correlative in nature [21,62]. Future investigations utilizing in vitro cell cultures or in vivo gene-targeted interventions (such as CRISPR/Cas9) are definitively required to establish causal links for these regulatory nodes in teleost growth. Finally, longitudinal studies integrating a wider array of high-density aquaculture variables will be essential to fully chart the temporal dynamics of chronic physiological adaptation.

## 5. Conclusions

**Figure 12:**
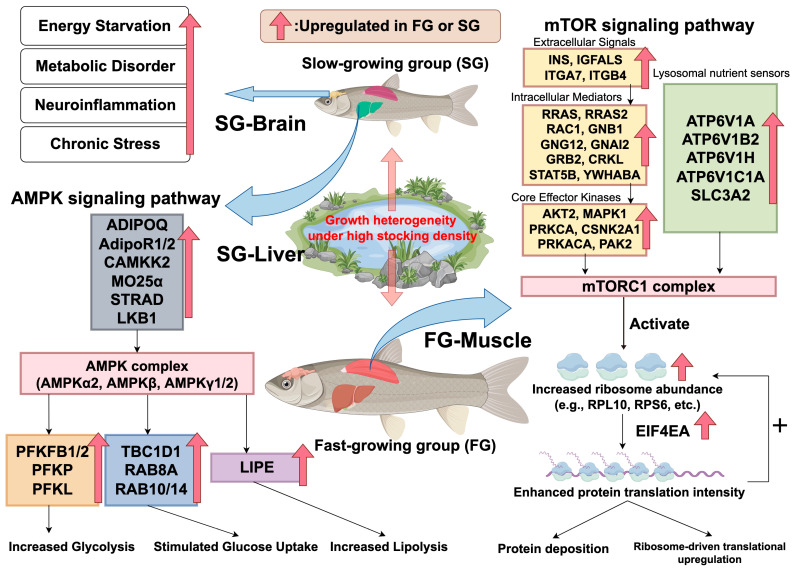
Perspectives on changes in the brain, muscle, and liver tissues between fast-growing (FG) and slow-growing (SG) grass carp under high stocking density.

The study suggests that growth divergence in grass carp is associated with a systemic decoupling of nutrient distribution and translation control. As shown in Figure 12, the fast-growing phenotype appears to be linked to post-transcriptional activation of the mTOR–ribosome axis in muscle, which may maximize protein deposition capacity, independent of transcript abundance. Conversely, slow growth appears to be constrained by three interconnected factors: central neuroimmune inflammation, a translational bottleneck in muscle, and an AMPK-mediated energy-wasting process in the liver. Together, these factors suggest a potential dissipation of systemic energy and deprivation of anabolic substrates for muscle growth. While this multi-organ post-transcriptional remodeling provides a systemic framework for understanding growth divergence, our findings primarily identify correlations; further functional studies are necessary to establish direct causal mechanisms.

## Figures and Tables

**Table 1:** Comparison of PRM and DIA quantification results. The ratio represents the expression fold change of the slow-growing group relative to the fast-growing group (SG/FG). “Only SG” indicates that the target molecule was exclusively detected in the SG group (i.e., the expression level is 0 in the FG group). PRM: parallel reaction monitoring; DIA: data-independent acquisition; FG: fast-growing; SG: slow-growing.

Protein Description	Symbol	Tissue	PRM Ratio (SG/FG)	DIA Ratio (SG/FG)
60S ribosomal protein L35a	rpl35a	Muscle	0.610	0.458058227
60S ribosomal protein L13	rpl13	Muscle	0.777	0.518517618
transferrin receptor 1a	TFRC	Muscle	0.716	0.706003693
eukaryotic translation initiation factor 3 subunit I	eif3i	Muscle	1.388	Only SG
parvalbumin-2	pvalb2	Muscle	0.633	0.517715427
TSC22 domain family protein 1 isoform X1	TSC22D1	Muscle	0.771	0.133316284
ras-related protein R-Ras2 isoform X1	RRAS2	Muscle	1.386	3.905915456
eukaryotic initiation factor 4A-III	eif4a3	Muscle	0.617	0.125688121
calcium/calmodulin-dependent protein kinase 2-like isoform X1	CAMKK2	Liver	1.295	22.580
5′-AMP-activated protein kinase catalytic subunit alpha-2	PRKAA2	Liver	1.014	Only SG
fructose-1,6-bisphosphatase 1b	FBP1	Liver	1.009	2.520
glycogen [starch] synthase, liver	GYS2	Liver	1.394	2.245
glycogen phosphorylase, liver form	Pygl	Liver	1.163	1.874
fatty acid synthase	FASN	Liver	1.047	2.168
insulin-like growth factor 2 mRNA-binding protein 1 isoform X1	igf2bp1	Liver	1.342	2.109
calcium-binding protein 39	CAB39	Liver	1.634	Only SG
proteasome subunit alpha type-3	Psma3	Liver	1.677	1.389
proteasome subunit alpha type-6a	PSMA6	Liver	1.215	1.585
proteasome subunit beta type-2	PSMB2	Liver	1.199	2.154

## Data Availability

The original contributions of this study are included in the article and Supplementary Materials; for further inquiries, please contact the corresponding authors.

## Supplementary Materials

The following supporting information can be downloaded at: https://www.mdpi.com/article/10.3390/ani16142205/s1, Figure S1: Overview of protein identification statistics and principal component analysis (PCA) across muscle, liver, and brain tissues; Figure S2: GSEA enrichment plots of proteomics data for pathways enriched in the fourth quadrant of the integrated transcriptomic and proteomic analysis in muscle tissue; Table S1: Summary of transcriptome sequencing data and read alignment rates; Table S2: Primer sequences used for quantitative real-time PCR (qPCR) analysis; Table S3: GSEA results of proteomics data for pathways enriched in the fourth quadrant of the integrated transcriptomic and proteomic analysis in muscle tissue; Table S4: The mRNA read count matrix and normalized expression levels across brain, liver, and muscle tissues. Table S5: The protein quantification matrix and differential expression levels across brain, liver, and muscle tissues.

## References

[1] FAO (2022). The State of World Fisheries and Aquaculture.

[2] Lu X., Chen H.-M., Qian X.-Q., Gui J.-F. (2020). Transcriptome Analysis of Grass Carp (*Ctenopharyngodon Idella*) between Fast- and Slow-Growing Fish. Comp. Biochem. Physiol. Part D Genom. Proteom..

[3] Ashley P.J. (2007). Fish Welfare: Current Issues in Aquaculture. Appl. Anim. Behav. Sci..

[4] Conte F.S. (2004). Stress and the Welfare of Cultured Fish. Appl. Anim. Behav. Sci..

[5] Ye W., Shi M., Chen S., Duan Y., Jiang Y., Cheng Y., Zhang W., Chen J., Wang Y., Xia X.-Q. (2023). Transcriptome Analysis Revealed the Existence of Family-Specific Regulation of Growth Traits in Grass Carp. Genomics.

[6] Causey D.R., Kim J.-H., Stead D.A., Martin S.A.M., Devlin R.H., Macqueen D.J. (2019). Proteomic Comparison of Selective Breeding and Growth Hormone Transgenesis in Fish: Unique Pathways to Enhanced Growth. J. Proteom..

[7] Liu Y., Beyer A., Aebersold R. (2016). On the Dependency of Cellular Protein Levels on mRNA Abundance. Cell.

[8] Conde-Sieira M., Soengas J.L. (2017). Nutrient Sensing Systems in Fish: Impact on Food Intake Regulation and Energy Homeostasis. Front. Neurosci..

[9] Polakof S., Panserat S., Soengas J.L., Moon T.W. (2012). Glucose Metabolism in Fish: A Review. J. Comp. Physiol. B.

[10] Canosa L.F., Chang J.P., Peter R.E. (2007). Neuroendocrine Control of Growth Hormone in Fish. Gen. Comp. Endocrinol..

[11] Bertucci J.I., Blanco A.M., Sundarrajan L., Rajeswari J.J., Velasco C., Unniappan S. (2019). Nutrient Regulation of Endocrine Factors Influencing Feeding and Growth in Fish. Front. Endocrinol..

[12] Perez É.S., Duran B.O.S., Zanella B.T.T., Dal-Pai-Silva M. (2023). Review: Understanding Fish Muscle Biology in the Indeterminate Growth Species Pacu (Piaractus Mesopotamicus). Comp. Biochem. Physiol. A Mol. Integr. Physiol..

[13] Johnston I.A. (2006). Environment and Plasticity of Myogenesis in Teleost Fish. J. Exp. Biol..

[14] Fraser K.P.P., Rogers A.D. (2007). Protein Metabolism in Marine Animals: The Underlying Mechanism of Growth. Adv. Mar. Biol..

[15] Seiliez I., Gabillard J.-C., Skiba-Cassy S., Garcia-Serrana D., Gutiérrez J., Kaushik S., Panserat S., Tesseraud S. (2008). An in Vivo and in Vitro Assessment of TOR Signaling Cascade in Rainbow Trout (*Oncorhynchus mykiss*). Am. J. Physiol.-Regul. Integr. Comp. Physiol..

[16] Wullschleger S., Loewith R., Hall M.N. (2006). TOR Signaling in Growth and Metabolism. Cell.

[17] Kamalam B.S., Medale F., Panserat S. (2017). Utilisation of Dietary Carbohydrates in Farmed Fishes: New Insights on Influencing Factors, Biological Limitations and Future Strategies. Aquaculture.

[18] Hardie D.G., Ross F.A., Hawley S.A. (2012). AMPK: A Nutrient and Energy Sensor That Maintains Energy Homeostasis. Nat. Rev. Mol. Cell Biol..

[19] Polakof S., Panserat S., Craig P.M., Martyres D.J., Plagnes-Juan E., Savari S., Aris-Brosou S., Moon T.W. (2011). The Metabolic Consequences of Hepatic AMP-Kinase Phosphorylation in Rainbow Trout. PLoS ONE.

[20] Herzig S., Shaw R.J. (2018). AMPK: Guardian of Metabolism and Mitochondrial Homeostasis. Nat. Rev. Mol. Cell Biol..

[21] Vogel C., Marcotte E.M. (2012). Insights into the Regulation of Protein Abundance from Proteomic and Transcriptomic Analyses. Nat. Rev. Genet..

[22] Maier T., Güell M., Serrano L. (2009). Correlation of mRNA and Protein in Complex Biological Samples. FEBS Lett..

[23] Chen S., Zhou Y., Chen Y., Gu J. (2018). Fastp: An Ultra-Fast All-in-One FASTQ Preprocessor. Bioinformatics.

[24] Langmead B., Salzberg S.L. (2012). Fast Gapped-Read Alignment with Bowtie 2. Nat. Methods.

[25] Kim D., Langmead B., Salzberg S.L. (2015). HISAT: A Fast Spliced Aligner with Low Memory Requirements. Nat. Methods.

[26] Pertea M., Pertea G.M., Antonescu C.M., Chang T.-C., Mendell J.T., Salzberg S.L. (2015). StringTie Enables Improved Reconstruction of a Transcriptome from RNA-Seq Reads. Nat. Biotechnol..

[27] Li B., Dewey C.N. (2011). RSEM: Accurate Transcript Quantification from RNA-Seq Data with or without a Reference Genome. BMC Bioinform..

[28] Love M.I., Huber W., Anders S. (2014). Moderated Estimation of Fold Change and Dispersion for RNA-Seq Data with DESeq2. Genome Biol..

[29] Benjamini Y., Hochberg Y. (1995). Controlling the False Discovery Rate: A Practical and Powerful Approach to Multiple Testing. J. R. Stat. Soc. Ser. B Stat. Methodol..

[30] Ashburner M., Ball C.A., Blake J.A., Botstein D., Butler H., Cherry J.M., Davis A.P., Dolinski K., Dwight S.S., Eppig J.T. (2000). Gene Ontology: Tool for the Unification of Biology. Nat. Genet..

[31] Kanehisa M. (2000). KEGG: Kyoto Encyclopedia of Genes and Genomes. Nucleic Acids Res..

[32] Fang Z., Liu X., Peltz G. (2023). GSEApy: A Comprehensive Package for Performing Gene Set Enrichment Analysis in Python. Bioinformatics.

[33] Subramanian A., Tamayo P., Mootha V.K., Mukherjee S., Ebert B.L., Gillette M.A., Paulovich A., Pomeroy S.L., Golub T.R., Lander E.S. (2005). Gene Set Enrichment Analysis: A Knowledge-Based Approach for Interpreting Genome-Wide Expression Profiles. Proc. Natl. Acad. Sci. USA.

[34] Livak K.J., Schmittgen T.D. (2001). Analysis of Relative Gene Expression Data Using Real-Time Quantitative PCR and the 2^−ΔΔCT^ Method. Methods.

[35] Tang R., Dodd A., Lai D., McNabb W.C., Love D.R. (2007). Validation of Zebrafish (*Danio rerio*) Reference Genes for Quantitative Real-Time RT-PCR Normalization. Acta Biochim. Biophys. Sin..

[36] Zhang J., Guo X., Han Z., Qu L., Xia T., Chen X., Xu J., Ding Z., Wei C., Cheng H. (2023). Comparative Analysis of Hepatopancreas RNA-Seq of Juvenile Grass Carp (*Ctenopharyngodon idella*) Fed Different Starch Diets. Fishes.

[37] Boshra H., Li J., Sunyer J.O. (2006). Recent Advances on the Complement System of Teleost Fish. Fish Shellfish Immunol..

[38] Schwarcz R., Bruno J.P., Muchowski P.J., Wu H.-Q. (2012). Kynurenines in the Mammalian Brain: When Physiology Meets Pathology. Nat. Rev. Neurosci..

[39] Evans R.M., Mangelsdorf D.J. (2014). Nuclear Receptors, RXR, and the Big Bang. Cell.

[40] Soengas J.L. (2014). Contribution of Glucose- and Fatty Acid Sensing Systems to the Regulation of Food Intake in Fish. A Review. Gen. Comp. Endocrinol..

[41] Volkoff H., Canosa L.F., Unniappan S., Cerdá-Reverter J.M., Bernier N.J., Kelly S.P., Peter R.E. (2005). Neuropeptides and the Control of Food Intake in Fish. Gen. Comp. Endocrinol..

[42] Spriggs K.A., Bushell M., Willis A.E. (2010). Translational Regulation of Gene Expression during Conditions of Cell Stress. Mol. Cell.

[43] Sweatt J.D. (2001). The Neuronal MAP Kinase Cascade: A Biochemical Signal Integration System Subserving Synaptic Plasticity and Memory. J. Neurochem..

[44] Furuhashi M., Hotamisligil G.S. (2008). Fatty Acid-Binding Proteins: Role in Metabolic Diseases and Potential as Drug Targets. Nat. Rev. Drug Discov..

[45] McArthur S., Cristante E., Paterno M., Christian H., Roncaroli F., Gillies G.E., Solito E. (2010). Annexin A1: A Central Player in the Anti-Inflammatory and Neuroprotective Role of Microglia. J. Immunol..

[46] Kültz D. (2005). Molecular and evolutionary basis of the cellular stress response. Annu. Rev. Physiol..

[47] Goodrich H.R., Wood C.M., Wilson R.W., Clark T.D., Last K.B., Wang T. (2024). Specific Dynamic Action: The Energy Cost of Digestion or Growth?. J. Exp. Biol..

[48] Wu G. (2009). Amino Acids: Metabolism, Functions, and Nutrition. Amino Acids.

[49] Brosnan J.T., Brosnan M.E. (2006). Branched-Chain Amino Acids: Enzyme and Substrate Regulation. J. Nutr..

[50] Suryawan A., Hawes J.W., Harris R.A., Shimomura Y., Jenkins A.E., Hutson S.M. (1998). A Molecular Model of Human Branched-Chain Amino Acid Metabolism. Am. J. Clin. Nutr..

[51] Magnoni L.J., Vraskou Y., Palstra A.P., Planas J.V. (2012). AMP-Activated Protein Kinase Plays an Important Evolutionary Conserved Role in the Regulation of Glucose Metabolism in Fish Skeletal Muscle Cells. PLoS ONE.

[52] Tian J., Lu X., Jiang M., Wu F., Liu W., Yu L., Wen H. (2020). AMPK Activation by Dietary AICAR Affects the Growth Performance and Glucose and Lipid Metabolism in Juvenile Grass Carp. Aquac. Nutr..

[53] Hardie D.G. (2011). AMP-Activated Protein Kinase—An Energy Sensor That Regulates All Aspects of Cell Function. Genes Dev..

[54] Biamonti G., Caceres J.F. (2009). Cellular Stress and RNA Splicing. Trends Biochem. Sci..

[55] Schiaffino S., Mammucari C. (2011). Regulation of Skeletal Muscle Growth by the IGF1-Akt/PKB Pathway: Insights from Genetic Models. Skelet. Muscle.

[56] Carter C.G., Houlihan D.F., Brechin J., McCarthy I.D. (1993). The Relationships between Protein Intake and Protein Accretion, Synthesis, and Retention Efficiency for Individual Grass Carp, *Ctenopharyngodon idella* (Valenciennes). Can. J. Zool..

[57] Dobly A., Martin S.A.M., Blaney S.C., Houlihan D.F. (2004). Protein Growth Rate in Rainbow Trout (Oncorhynchus Mykiss) Is Negatively Correlated to Liver 20S Proteasome Activity. Comp. Biochem. Physiol. Part A Mol. Integr. Physiol..

[58] Tidball J.G. (2005). Inflammatory Processes in Muscle Injury and Repair. Am. J. Physiol.-Regul. Integr. Comp. Physiol..

[59] Chazaud B. (2014). Macrophages: Supportive Cells for Tissue Repair and Regeneration. Immunobiology.

[60] Grefte S., Kuijpers-Jagtman A.M., Torensma R., Von Den Hoff J.W. (2007). Skeletal Muscle Development and Regeneration. Stem Cells Dev..

[61] Yu E.-M., Zhang H.-F., Li Z.-F., Wang G.-J., Wu H.-K., Xie J., Yu D.-G., Xia Y., Zhang K., Gong W.-B. (2017). Proteomic Signature of Muscle Fibre Hyperplasia in Response to Faba Bean Intake in Grass Carp. Sci. Rep..

[62] Schwanhäusser B., Busse D., Li N., Dittmar G., Schuchhardt J., Wolf J., Chen W., Selbach M. (2011). Global Quantification of Mammalian Gene Expression Control. Nature.

[63] Boisclair Y., Rhoads R., Ueki I., Wang J., Ooi G. (2001). The Acid-Labile Subunit (ALS) of the 150 kDa IGF-Binding Protein Complex: An Important but Forgotten Component of the Circulating IGF System. J. Endocrinol..

[64] Nicklin P., Bergman P., Zhang B., Triantafellow E., Wang H., Nyfeler B., Yang H., Hild M., Kung C., Wilson C. (2009). Bidirectional Transport of Amino Acids Regulates mTOR and Autophagy. Cell.

[65] Zoncu R., Bar-Peled L., Efeyan A., Wang S., Sancak Y., Sabatini D.M. (2011). mTORC1 Senses Lysosomal Amino Acids Through an Inside-Out Mechanism That Requires the Vacuolar H+-ATPase. Science.

[66] Sancak Y., Bar-Peled L., Zoncu R., Markhard A.L., Nada S., Sabatini D.M. (2010). Ragulator-Rag Complex Targets mTORC1 to the Lysosomal Surface and Is Necessary for Its Activation by Amino Acids. Cell.

[67] Mendoza M.C., Er E.E., Blenis J. (2011). The Ras-ERK and PI3K-mTOR Pathways: Cross-Talk and Compensation. Trends Biochem. Sci..

[68] Richter J.D., Sonenberg N. (2005). Regulation of Cap-Dependent Translation by eIF4E Inhibitory Proteins. Nature.

[69] Deal C.K., Volkoff H. (2020). The Role of the Thyroid Axis in Fish. Front. Endocrinol..

[70] Power D.M., Llewellyn L., Faustino M., Nowell M.A., Björnsson B.T., Einarsdottir I.E., Canario A.V.M., Sweeney G.E. (2001). Thyroid Hormones in Growth and Development of Fish. Comp. Biochem. Physiol. Part C Toxicol. Pharmacol..

[71] Brown M.C., Turner C.E. (2004). Paxillin: Adapting to Change. Physiol. Rev..

