# Supplementary material for: Integrated Transcriptomic and Proteomic Analysis Unveils the Multi-Organ Regulatory Mechanisms of Growth Divergence in Grass Carp (Ctenopharyngodon idella)
Source: Animals (Basel). 2026 Jul 15;16(14):2205. doi: 10.3390/ani16142205 (PMC13403668; doi:10.3390/ani16142205)
Supplement: Supplementary file 1 [file animals-16-02205-s001.zip › Supplementary Figures.pdf]

Supplementary Materials: Overview of protein identification statistics and principal component analysis (PCA) across muscle, liver, and brain tissues, as well as GSEA enrichment plots of proteomics data for pathways enriched in the fourth quadrant of the integrated transcriptomic and proteomic analysis in muscle tissue.

Manuscript title: Integrated Transcriptomic and Proteomic Analysis Unveils the Multi-Organ Regulatory Mechanisms of Growth Divergence in Grass Carp (*Ctenopharyngodon idella*)

1. Overview of protein identification statistics and principal component analysis (PCA) across muscle, liver, and brain tissues

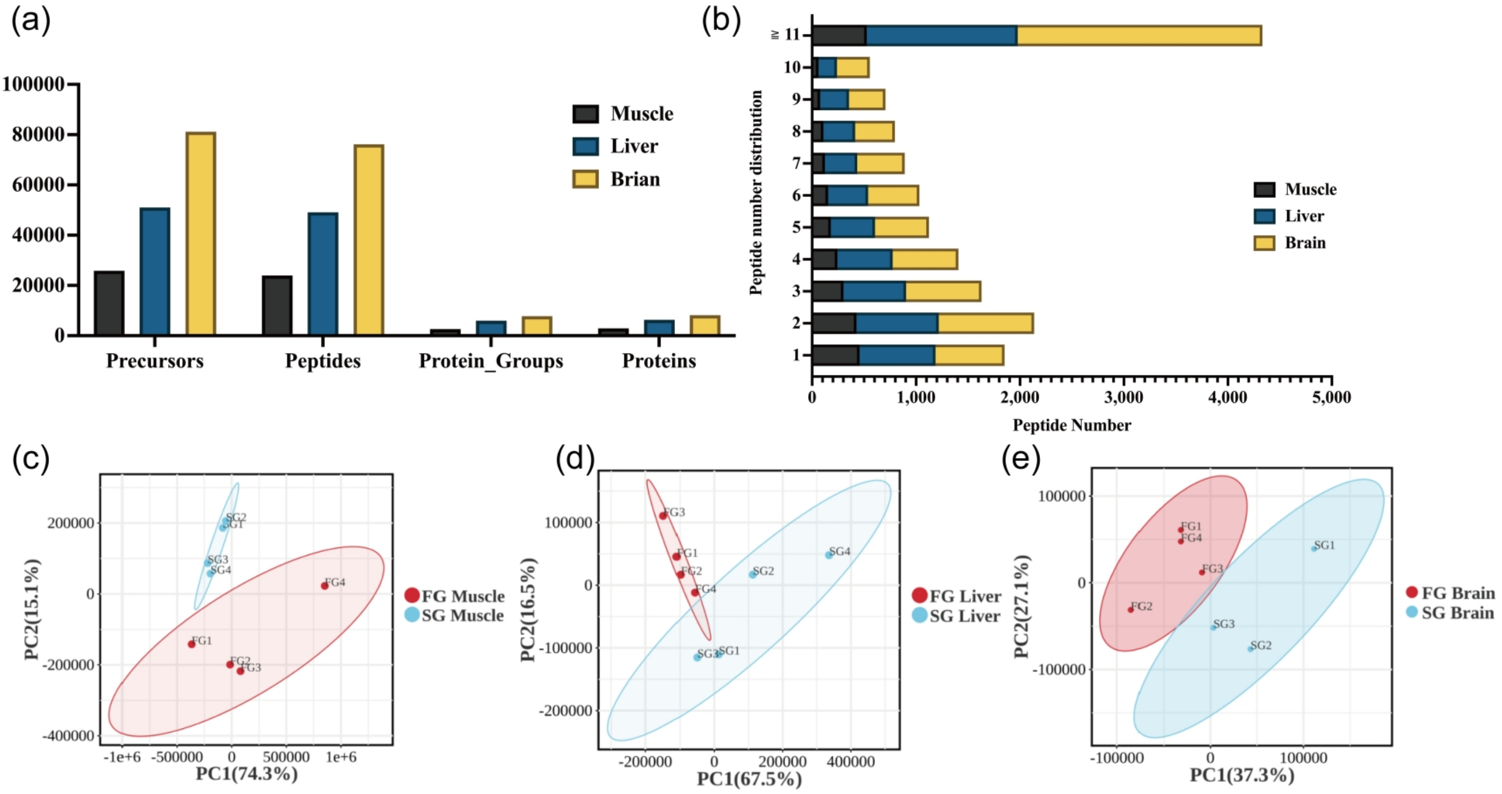

Figure S1. Proteome analysis between the FG and SG groups. (a) Statistical results of protein and peptide identification. (b) Distribution of peptide numbers per protein. (c–e) Principal Component Analysis (PCA) of samples from the FG and SG groups in (c) muscle, (d) liver, and (e) brain tissues.

2. GSEA enrichment plots of proteomics data for pathways enriched in the fourth quadrant of the integrated transcriptomic and proteomic analysis in muscle tissue

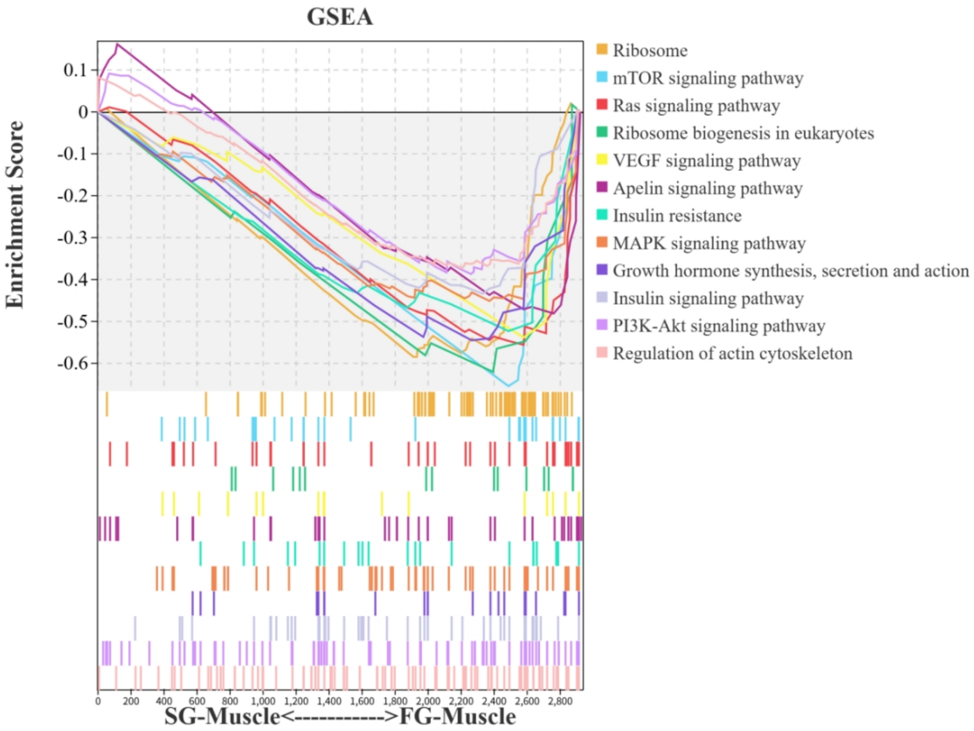

Figure S2. Gene Set Enrichment Analysis (GSEA) of growth-related signaling pathways in grass carp skeletal muscle. The enrichment plots display the coordinated post-transcriptional activation of core anabolic pathways in the fast-growing (FG) group compared to the slow-growing (SG) group. Pathways were identified from Quadrant IV of the nine-quadrant integrated transcriptomic and proteomic analysis, representing molecules with stable mRNA levels but significantly upregulated protein abundance.
